# Disproportionate left atrial myopathy in heart failure with preserved ejection fraction among participants of the PROMIS-HFpEF study

**DOI:** 10.1038/s41598-021-84133-9

**Published:** 2021-03-01

**Authors:** Ravi B. Patel, Carolyn S. P. Lam, Sara Svedlund, Antti Saraste, Camilla Hage, Ru-San Tan, Lauren Beussink-Nelson, Jasper Tromp, Cynthia Sanchez, Joyce Njoroge, Stanley A. Swat, Ulrika Ljung Faxén, Maria Lagerstrom Fermer, Ashwin Venkateshvaran, Li-Ming Gan, Lars H. Lund, Sanjiv J. Shah

**Affiliations:** 1 Division of Cardiology, Department of Medicine, Northwestern University Feinberg School of Medicine, Chicago, IL USA; 2 National Heart Centre Singapore, Duke-National University of Singapore, Singapore, Singapore; 3 University Medical Centre, Groningen, The Netherlands; 4 The George Institute for Global Health, Sydney, Australia; 5 Department of Clinical Physiology, Institute of Medicine, Sahlgrenska University Hospital, University of Gothenburg, Gothenburg, Sweden; 6 Heart Center, Turku University Hospital, University of Turku, Turku, Finland; 7 Department of Medicine, Cardiology Unit and Heart and Vascular Theme, Karolinska Institutet, Stockholm, Sweden; 8 Early Clinical Development, Research and Early Development, Cardiovascular, Renal and Metabolism (CVRM), BioPhamaceuticals R&D, AstraZeneca, Gothenburg, Sweden; 9 Department of Molecular and Clinical Medicine, Institute of Medicine, Sahlgrenska Academy At the University of Gothenburg, Gothenburg, Sweden; 10 Department of Cardiology, Sahlgrenska University Hospital, Gothenburg, Sweden; 11 Division of Cardiology, Department of Medicine, Northwestern University Feinberg School of Medicine, 676 N. St Clair St, Suite 600, Chicago, IL 60611 USA

**Keywords:** Cardiovascular diseases, Heart failure

## Abstract

Impaired left atrial (LA) function in heart failure with preserved ejection fraction (HFpEF) is associated with adverse outcomes. A subgroup of HFpEF may have LA myopathy out of proportion to left ventricular (LV) dysfunction; therefore, we sought to characterize HFpEF patients with disproportionate LA myopathy. In the prospective, multicenter, Prevalence of Microvascular Dysfunction in HFpEF study, we defined disproportionate LA myopathy based on degree of LA reservoir strain abnormality in relation to LV myopathy (LV global longitudinal strain [GLS]) by calculating the residuals from a linear regression of LA reservoir strain and LV GLS. We evaluated associations of disproportionate LA myopathy with hemodynamics and performed a plasma proteomic analysis to identify proteins associated with disproportionate LA myopathy; proteins were validated in an independent sample. Disproportionate LA myopathy correlated with better LV diastolic function but was associated with lower stroke volume reserve after passive leg raise independent of atrial fibrillation (AF). Additionally, disproportionate LA myopathy was associated with higher pulmonary artery systolic pressure, higher pulmonary vascular resistance, and lower coronary flow reserve. Of 248 proteins, we identified and validated 5 proteins (involved in cardiomyocyte stretch, extracellular matrix remodeling, and inflammation) that were associated with disproportionate LA myopathy independent of AF. In HFpEF, LA myopathy may exist out of proportion to LV myopathy. Disproportionate LA myopathy is a distinct HFpEF subtype associated with worse hemodynamics and a distinct proteomic signature, independent of AF.

## Introduction

While traditionally viewed as a syndrome of left ventricular (LV) diastolic dysfunction, heart failure (HF) with preserved ejection fraction (HFpEF) may also be characterized by adverse changes to left atrial (LA) myocardial structure and function, resulting in LA myopathy^1,2^. Speckle-tracking strain echocardiography, a sensitive measure of myocardial performance, can quantify severity of LA myopathy through assessment of abnormalities in intrinsic LA reservoir function^3,4^. Importantly, LA myopathy, as defined by reduced LA reservoir strain, appears to be a stronger predictor of mortality among patients with established HFpEF than indices of ventricular structure or function^5^. The spectrum and degree of LA myopathy in HFpEF are wide, as it may develop secondary to LV myopathy (secondary to high LV filling pressures), concurrent with LV myopathy as part of similar pathophysiology affecting both LA and LV, or out of proportion to LV myopathy (due to intrinsic abnormalities of the LA and/or atrial fibrillation [AF])^6,7^.

Despite the prognostic implications of LA dysfunction, the clinical, echocardiographic, and hemodynamic profiles of HFpEF based on degree of LA myopathy in relation to LV myopathy have not been well defined. Thus, our primary goal was to evaluate for the presence of “disproportionate LA myopathy” in HFpEF, and comprehensively characterize this previously undefined phenotype. As such, we aimed to (1) phenotype a diverse HFpEF cohort based on degree of LA myopathy as compared with LV myopathy; (2) evaluate the associations of disproportionate LA myopathy with hemodynamics and coronary microvascular dysfunction; and (3) evaluate the proteomic profile of disproportionate LA myopathy in participants enrolled in the multicenter, prospective Prevalence of Microvascular Dysfunction in HFpEF (PROMIS-HFpEF) study. We hypothesized that LA myopathy may exist out of proportion to LV myopathy and is associated with abnormal hemodynamics along with distinct clinical and proteomic profiles, independent of AF.

## Results

### Clinical, laboratory, and echocardiographic variables associated with disproportionate LA myopathy

**Figure 1:**
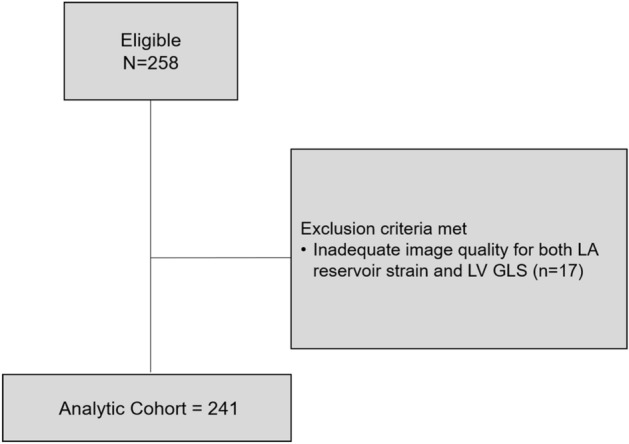
STROBE flow diagram for study inclusion.

**Figure 2:**
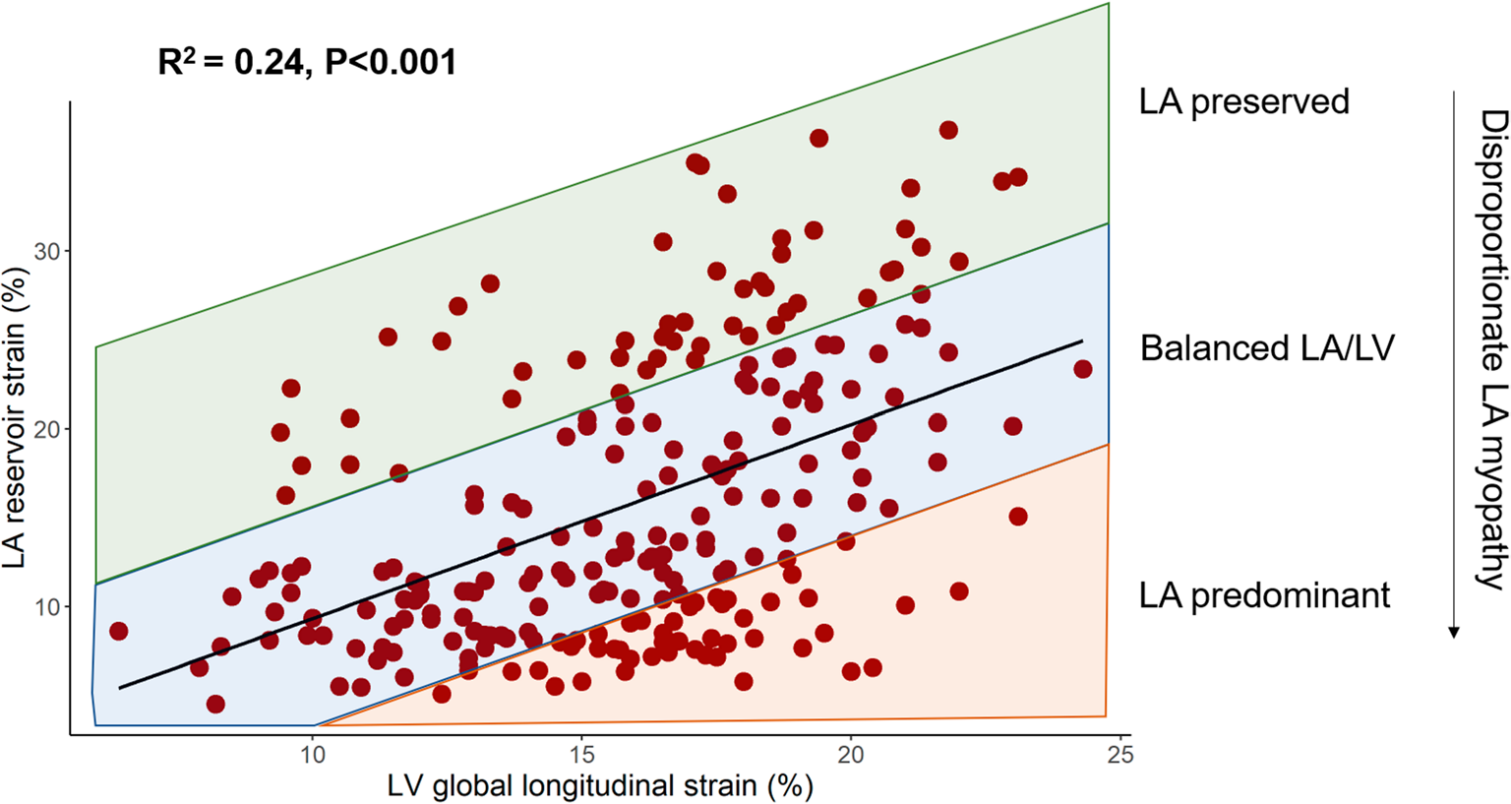
Association of LV global longitudinal strain with LA reservoir strain. Degree of LA myopathy (i.e. disproportionate LA myopathy) was defined as studentized residual values derived from the linear regression model of LV GLS and LA reservoir strain. Lower residual values thus correspond to disproportionately greater LA myopathy compared with LV myopathy. *GLS* global longitudinal strain, *LA* left atrial, *LV* left ventricular.

This was a secondary analysis of the PROMIS-HFpEF cohort, which was an international study designed to evaluate the prevalence and correlates of coronary microvascular dysfunction in chronic HFpEF. We defined disproportionate LA myopathy based on degree of LA reservoir strain abnormality in relation to LV myopathy (LV global longitudinal strain [GLS]) by calculating the residuals from a linear regression of LA reservoir strain and LV GLS. Of 258 participants in the PROMIS cohort, 241 had adequate images for LA reservoir strain and LV GLS at baseline and were thus included in our analyses (Fig. 1). There was a significant but modest association of LV GLS with LA reservoir strain (Fig. 2). The clinical and laboratory characteristics of the analytic cohort are shown in Table 1. Characteristics significantly associated with disproportionate LA myopathy included older age, white race, AF, and chronic kidney disease. Higher levels of diastolic blood pressure, N-terminal pro-B-type natriuretic peptide (NT-proBNP), and troponin T, and lower body mass index (BMI) were also significantly associated with disproportionate LA myopathy. Findings were consistent upon comparing participants by those with studentized residual values < 0 (indicative of LA myopathy) vs. those with residuals > 0 (Supplemental Table S1).

**Table 1:** Characteristics associated with disproportionate left atrial myopathy in PROMIS-HFpEF.

Characteristic	PROMIS-HFpEF cohort (n = 241)	β coefficient (SE)	P value
Age, year	75 (70–81)	0.04 (0.01)	< 0.001
Male sex, n (%)	107 (45)	0.19 (0.13)	0.14
White race, n (%)	209 (87)	0.84 (0.30)	0.006
NYHA class III/IV	61 (25.6)	− 0.006 (0.15)	0.97
**Comorbidities**			
Hypertension, n (%)	198 (83)	− 0.09 (0.17)	0.59
Diabetes, n (%)	63 (26)	0.09 (0.15)	0.54
Hyperlipidemia, n (%)	128 (53)	− 0.26 (0.13)	0.04
Atrial fibrillation, n (%)	99 (41.1)	1.20 (0.11)	< 0.001
ASCVD, n (%)	100 (42)	3.8 × 10^–3^ (0.13)	0.98
Never Smokers	77 (32)	− 0.13 (0.14)	0.38
Chronic kidney disease, n (%)	126 (52)	0.27 (0.13)	0.04
Pacemaker or ICD, n (%)	41 (17)	− 0.05 (0.17)	0.77
**Vital signs, physical characteristics, and laboratory data**			
Body mass index, kg/m^2^	28.0 (24.4–32.5)	− 0.02 (0.01)	0.008
Heart rate, bpm	68 (60–78)	0.006 (0.005)	0.23
SBP, mmHg	139 (127–152)	0.002 (0.003)	0.57
DBP, mmHg	77 (68–85)	0.02 (0.01)	0.002
Waist circumference, cm	100 (89–113)	− 0.002 (0.004)	0.61
NT-proBNP, pg/mL	960 (365–1770)	0.36 (0.05)	< 0.001
Sodium, mEq/L	140 (138–142)	0.04 (0.02)	0.12
Potassium, mEq/L	4.2 (3.9–4.5)	0.26 (0.15)	0.09
Creatinine, mg/dL	1.06 (0.86–1.35)	0.31 (0.20)	0.12
Glomerular filtration rate, mL/min/1.73 m^2^	58.7 (45.6–69.7)	− 0.29 (0.18)	0.12
Troponin T, ng/mL	13.0 (10.0–21.1)	0.17 (0.06)	0.007
Hemoglobin A1c, %	41 (38–49)	0.08 (0.31)	0.80
Urine albumin-to-creatinine ratio, mg/g	3.1 (1.3–9.7)	0.13 (0.07)	0.07
6-min walk distance, m	332 (210–412)	− 0.15 (0.12)	0.21
Change in heart rate from baseline in response to adenosine, bpm	17 (9–28)	− 0.002 (0.004)	0.62
**Medications**			
Loop diuretic, n (%)	130 (54)	0.01 (0.13)	0.13
Thiazide diuretic, n (%)	26 (11)	− 0.13 (0.21)	0.54
Mineralocorticoid receptor antagonist, n (%)	69 (29)	− 0.06 (0.14)	0.67
ACE inhibitor, n (%)	71 (30)	0.07 (0.14)	0.62
ARB, n (%)	102 (42)	0.03 (0.13)	0.80

Continuous variables are reported as median (interquartile range).

*ACE* angiotensin converting enzyme, *ARB* angiotensin receptor blocker, *ASCVD* atherosclerotic vascular disease, *DBP* diastolic blood pressure, *GFR* glomerular filtrate rate; *ICD* implantable cardioverter-defibrillator, *NT-proBNP* N-terminal pro-B-type natriuretic peptide, *NYHA* New York Heart Association, *SBP* systolic blood pressure, *UACR* urinary albumin-to-creatinine ratio.

**Figure 3:**
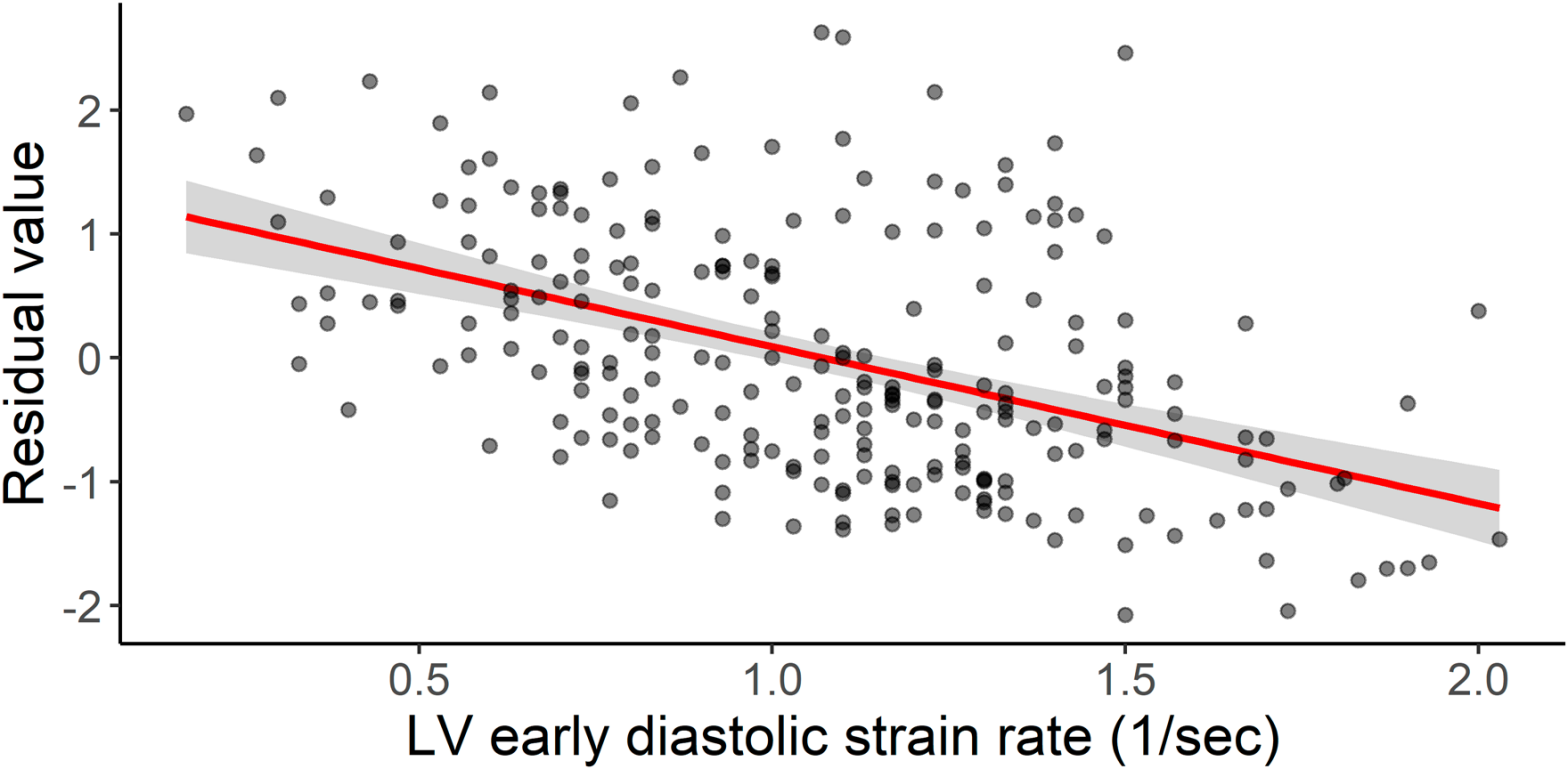
Association of higher early diastolic strain rate with disproportionate LA myopathy. Shown is the relationship between early diastolic strain rate and the residual values of the regression model between LV GLS and LA reservoir strain (lower residual values indicate disproportionate LA myopathy). The red line represents the linear regression model between the 2 variables. Higher early diastolic strain rate (i.e. better diastolic function) was associated with disproportionate LA myopathy (β coefficient: − 1.26, SE: 0.15, P < 0.001).

There were several echocardiographic and hemodynamic correlates of disproportionate LA myopathy (Table 2). Structural predictors of disproportionate LA myopathy included smaller LV end diastolic volume, and larger LA and right atrial (RA) size. LV functional predictors of disproportionate LA myopathy included higher e’ tissue velocities and early diastolic strain rate (Fig. 3). Notably, LV systolic function indices were not associated with disproportionate LA myopathy. Worse right ventricular (RV) systolic function as measured by tricuspid annular plane systolic excursion (TAPSE) and RV free wall strain was associated with disproportionate LA myopathy. Hemodynamic correlates of disproportionate LA myopathy included lower stroke volume/cardiac index, higher pulmonary artery systolic pressure, and pulmonary vascular resistance. Finally, disproportionate LA myopathy was associated with lower coronary flow reserve (CFR) (Supplemental Fig. S1).

**Table 2:** Echocardiographic variables associated with disproportionate left atrial myopathy.

Echocardiographic variable	PROMIS-HFpEF cohort (n = 241)	β coefficient (SE)	P value
**Left heart structure/function**			
LV end-diastolic volume index, mL/m^2^	41.1 (33.8–50.1)	− 0.55 (0.24)	0.02
LV end-systolic volume index, mL/m^2^	16.5 (12.5–21.4)	− 0.24 (0.17)	0.15
LV mass index, g/m^2^	102.5 (83.5–124.5)	0.25 (0.22)	0.27
Relative wall thickness	0.45 (0.40–0.52)	0.17 (0.34)	0.62
LV ejection fraction, %	60 (55–64)	0.13 (0.43)	0.77
LA volume index, mL/m^2^	38.4 (30.7–44.8)	1.62 (0.17)	< 0.001
e' septal velocity, cm/s	6.8 (5.3–8.3)	0.09 (0.02)	0.001
e' lateral velocity, cm/s	9.7 (7.8–12.1)	0.11 (0.02)	< 0.001
Average E/e' ratio	12.2 (9.3–15.9)	− 0.03 (0.17)	0.87
Early diastolic strain rate, 1/s	1.10 (0.78–1.33)	1.26 (0.15)	< 0.001
LV global longitudinal strain, %	16.5 (13.3–18.5)	1.4 × 10–4 (0.02)	0.99
LA reservoir strain, %	13.1 (9.2–22.2)	− 0.11 (0.004)	< 0.001
**Right heart structure/function**			
RV end-diastolic area, cm^2^	18.5 (15.0–22.3)	0.02 (0.01)	0.06
Right atrial area, cm^2^	19.5 (15.9–24.6)	0.07 (0.01)	< 0.001
TAPSE, cm	1.8 (1.6–2.1)	− 0.73 (0.17)	< 0.001
RV free wall strain, %	21.6 (17.8–25.4)	− 0.04 (0.01)	0.001
**Hemodynamics**			
PASP, mmHg	42 (35–51)	0.02 (0.005)	< 0.001
Pulmonary vascular resistance, WU	2.2 (1.7–2.8)	1.17 (0.19)	< 0.001
Stroke volume, mL	72 (54–85)	− 1.05 (0.19)	< 0.001
Cardiac index, L/min/m^2^	2.40 (2.00–2.86)	− 0.36 (0.09)	< 0.001
CFR	2.08 (1.78–2.50)	− 0.42 (0.13)	0.002

Continuous variables are reported as median (interquartile range).

*CFR* coronary flow reserve, *LA* left atrial, *LV* left ventricular, *PASP* pulmonary artery systolic pressure, *RV* right ventricular, *TAPSE* tricuspid annular plane systolic excursion.

### Association of disproportionate LA myopathy with hemodynamics

Upon examination as a continuous variable on linear regression analysis, disproportionate LA myopathy was significantly associated with lower resting stroke volume (SV) after multivariable adjustment (β-coefficient per 1-SD lower studentized residuals: − 6.8 [95% CI − 10.2, − 3.4] mL; P < 0.001; Table 3), including for AF. These findings were consistent on sensitivity analysis further adjusting for New York Heart Association (NYHA) class, 6-min walk distance, and heart rate response to adenosine infusion (Supplemental Table S2).

**Table 3:** Association of disproportionate left atrial myopathy with stroke volume and stroke volume reserve (after passive leg raise maneuver).

	β-coefficient per 1-unit decrease in studentized residual (95% CI)	P value
**Resting SV**		
Model 1^a^	− 5.2 (− 8.0, − 2.4)	< 0.001
Model 2^b^	− 6.8 (− 10.2, − 3.4)	< 0.001
**Change in SV after leg raise**^c^		
Model 1^a^	− 1.5 (− 3.0, − 0.04)	0.04
Model 2^b^	− 2.2 (− 4.2, − 0.2)	0.03

*BMI* body mass index, *GLS* global longitudinal strain, *LA* left atrial, *LAV* left atrial volume, *LV* left ventricular, *NT-proBNP* N-terminal pro-B-type natriuretic peptide, *SV* stroke volume.

^a^Adjusted for enrollment site, age, sex, race, BMI, hypertension, diabetes, creatinine, and average E/e′.

^b^Adjusted for Model 1 covariates plus LV GLS, LAV, NT-proBNP, and AF.

^c^All models adjusted for resting SV.

There was a wide variation in change in SV after passive leg raise (i.e., intravascular volume challenge) across the PROMIS cohort (range − 40.2 mL to + 39.6 mL; Supplemental Fig. S2). After multivariable adjustment, disproportionate LA myopathy was associated with decrease in SV after intravascular volume challenge (β-coefficient per 1-SD lower studentized residuals: − 2.2 [95% CI − 4.2, − 0.2] mL; P = 0.03; Table 3), independent of AF. These findings were consistent on sensitivity analysis (Supplemental Table S2).There was no interaction by AF on the association of disproportionate LA myopathy with resting SV (P_interaction_ = 0.09) or change in SV after passive leg raise maneuver (P_interaction_ = 0.80).

### Association of plasma proteins with disproportionate LA myopathy

**Figure 4:**
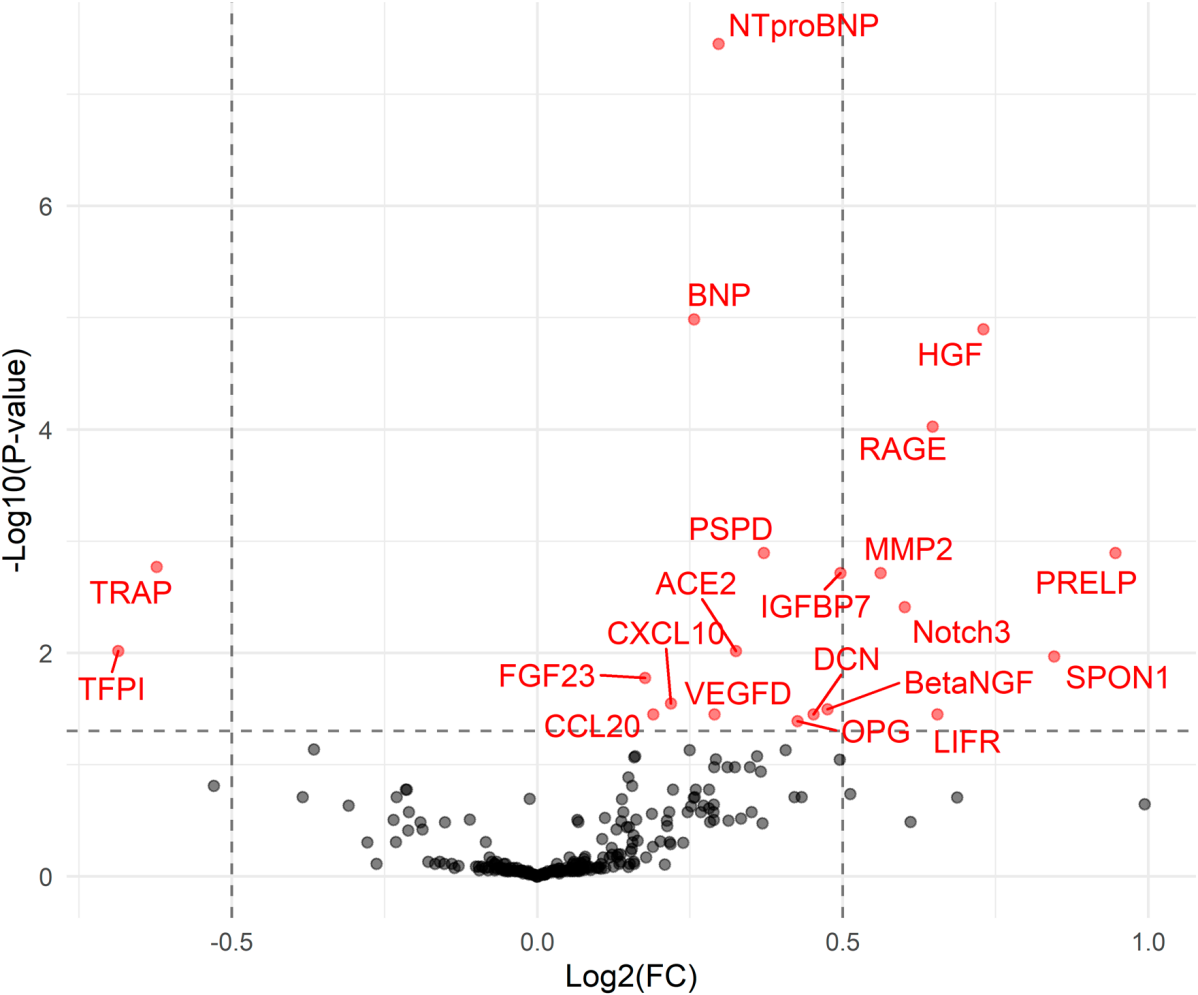
Association of plasma proteins with disproportionate LA myopathy in PROMIS-HFpEF. The volcano plot shows proteins positively and negatively associated with disproportionate LA myopathy. The y-axis represents false-discovery rate adjusted P-values. There were 21 proteins associated with disproportionate LA myopathy (red) after adjustment for multiple comparisons.

Of the 248 candidate proteins, there were 21 proteins significantly associated with disproportionate LA myopathy in PROMIS-HFpEF cohort after controlling for multiple comparisons (Fig. 4, Supplemental Table S3). In the Northwestern HFpEF validation cohort, 13 of the 21 proteins were also significantly associated with disproportionate LA myopathy (Supplemental Table S3). Of the 13 proteins demonstrating associations on univariate analysis, 5 distinct proteins remained significantly associated with disproportionate LA myopathy after adjustment for age, sex, and AF in both PROMIS and Northwestern HFpEF cohorts (Table 4). These 5 proteins have previously demonstrated pathway associations with cardiomyocyte stretch, collagen regulation, and inflammation (Supplemental Figs. S5–S9).

**Table 4:** Plasma proteins associated with disproportionate LA myopathy on multivariable linear regression in both PROMIS and Northwestern cohorts (validation).

Protein	PROMIS-HFpEF (n = 241)			Northwestern HFpEF cohort (n = 117)		
	β-coefficient	SE	P value*	β-coefficient	SE	P value*
NT-proBNP^a^	0.12	0.04	0.005	0.25	0.07	0.0006
BNP^a^	0.11	0.04	0.007	0.20	0.06	0.003
PRELP	0.52	0.18	0.006	1.00	0.40	0.01
TRAP	− 0.29	0.13	0.03	− 0.37	0.18	0.04
VEGFD	0.24	0.07	0.002	0.52	0.20	0.009
DCN	0.25	0.12	0.04	0.56	0.25	0.03

*False discovery rate-adjusted values. Adjusted for age, sex, and AF.

^a^NT-proBNP and BNP were categorized as 1 distinct protein given BNP is a product of cleavage of NT-proBNP.

*BNP* B-type natriuretic peptide, *DCN* decorin, *NT-proBNP* N-terminal pro-B-type natriuretic peptide, *PRELP* proline-arginine-rich end leucine-rich repeat protein, *TRAP* tartrate-resistant acid phosphatase, *VEGFD* vascular endothelial growth factor D.

### Comparison of plasma proteins associated with disproportionate LA myopathy and atrial fibrillation

Of the 248 candidate proteins, there were 11 proteins significantly associated with AF in PROMIS-HFpEF cohort after controlling for multiple comparisons (Supplemental Fig. S3). Of these 11 proteins, only 3 were validated in the Northwestern HFpEF cohort (Supplemental Table S4). Upon comparison of validated proteins associated with disproportionate LA myopathy (13 proteins) and AF (3 proteins), 10 proteins were uniquely associated with disproportionate LA myopathy and not AF (Supplemental Fig. S4). There were no proteins that were only associated with AF and not disproportionate LA myopathy (Supplemental Table S4, Fig. S4).

## Discussion

In this prospective, multicenter study of an international and rigorously-phenotyped HFpEF cohort, we characterized participants based on degree of LA myopathy as compared with LV myopathy. Despite favorable diastolic function, disproportionate LA myopathy was associated with adverse RV function and elevated pulmonary vascular resistance. Additionally, disproportionate LA myopathy was associated with diminished SV and SV reserve, independent of AF. There was a significant correlation between coronary microvascular dysfunction as measured by CFR and disproportionate LA myopathy. Finally, we identified and validated several plasma proteins that were associated with disproportionate LA myopathy independent of clinical characteristics including AF. We demonstrate that the proteome of disproportionate LA myopathy is distinct from that of AF. In aggregate, our findings identify and characterize a distinct HFpEF phenotype of disproportionate LA myopathy that cannot be fully explained by the presence of comorbid AF. Ultimately, HFpEF patients with disproportionate LA myopathy may ultimately benefit from targeted therapies to alleviate symptoms and improve outcomes.

Reduced LA reservoir strain has shown promise in identifying a high-risk HFpEF cohort. Indeed, LA reservoir strain is associated with adverse clinical outcomes in HFpEF^5,8,9^. Impaired LA reservoir strain has been more closely associated with poor prognosis in HFpEF than measures of LV function^5^. Furthermore, reduced LA reservoir strain has recently been associated with increased pulmonary capillary wedge pressure with exercise and lower peak oxygen consumption on cardiopulmonary exercise testing^6,10–12^. Despite this evidence, there has been some concern that reduced LA reservoir strain may simply represent a marker of global LV dysfunction, as there is a modest correlation between LA and LV strain in HFpEF^13^, and the association between LA reservoir strain and adverse outcomes may be explained by severity of LV myopathy, as measured by reduced LV strain^9,14^.

Our study is the first, to our knowledge, to define HFpEF based on relative degree of LA myopathy compared with LV myopathy to understand the degree to which disproportionate LA myopathy represents a unique phenotype of HFpEF. Disproportionate LA myopathy appears to have a truly “LA-specific” echocardiographic signature; disproportionate LA myopathy was associated with favorable diastolic function (higher e’ tissue velocity and early diastolic strain rate). Thus, our findings suggest LA myopathy may not simply be a consequence of LV disease, but rather represents a unique pathophysiology among patients with HFpEF. Despite favorable diastolic function, disproportionate LA myopathy correlated with a worse hemodynamic profile, highlighted by both higher pulmonary artery systolic pressure and pulmonary vascular resistance along with lower SV. Indeed, disproportionate LA myopathy was independently associated with reduced SV, even after adjustment for sensitive measures of LV function (LV GLS), LA dilation, biomarkers of HF severity (NT-proBNP), and AF, suggesting that LA myopathy is a distinct entity and not simply a marker of either more advanced HFpEF or the presence of AF.

Reduced LA function carries important implications regarding the ability of HFpEF patients to withstand intravascular volume challenges. Poor LA mechanical function in response to a passive leg raise maneuver has demonstrated adequate predictive capability in diagnosing HFpEF from hypertensive controls^15^. In our study, disproportionate LA myopathy was independently associated with inability to augment SV in response to a passive leg raise maneuver. Thus, in HFpEF, preserved LA function appears to play a crucial role in the ability to tolerate volume challenges through increased compliance to augment SV, which may be especially important during exertion or at times of stress.

While improvement in LA function may result in reduced congestive symptoms in a tenuous subset of the HFpEF cohort, identification of the specific HFpEF subgroup who may benefit from tailored, “LA-specific” therapies has proven challenging. Our study supports a novel method to identify HFpEF patients with disproportionate LA myopathy who may experience the most benefit from therapies to improve LA function. Patients with disproportionate LA myopathy may be considered as an unique population for trials specifically targeting the LA, such as the Reduce Elevated Left Atrial Pressure in Patients with Heart Failure II (REDUCE-LAP II; NCT03088033) trial, a phase III, randomized, sham-control trial of an interatrial shunt device, which has been shown to reduce pulmonary capillary wedge pressure during exercise in a mechanistic phase II trial^16^.

The unique design of PROMIS-HFpEF allowed for insight into mechanisms responsible for LA myopathy. In our study, there was a significant, modest correlation between disproportionate LA myopathy and CFR, suggesting that LA function has a relationship with coronary microvascular dysfunction. It is possible the correlation between disproportionate LA myopathy and CFR may be explained by lower coronary perfusion pressure in the setting of increased LV filling pressures. Additionally, we identified and validated 5 distinct plasma proteins that were associated with disproportionate LA myopathy independent of age, sex, and even AF. All validated proteins are each linked to the LA specifically, and thus provide potential insight into the pathophysiology and potential treatment targets for LA dysfunction. For example, as compared with the LV, BNP gene expression and tissue BNP levels are markedly upregulated within the LA selectively in the setting of early LV dysfunction^17^. Proline-arginine-rich end leucine-rich repeat protein (PRELP) and decorin both belong to a broader family of proteoglycans that regulates collagen formation, which has been implicated in the pathogenesis of AF and may regulate growth factors involved in LA hypertrophy^18^. Vascular endothelial growth factor D is strongly linked to AF development^19^. Finally, higher levels of tartrate-resistant acid phosphatase (TRAP) were associated with less LA myopathy, which may be due to TRAP’s protective role as a negative regulator of the inflammatory response and superoxide generation^20^.

In our study, AF was associated with disproportionate LA myopathy. It is possible that disproportionate LA myopathy is a consequence of AF itself. However, we have previously demonstrated that LA myopathy, as measured by LA reservoir strain, precedes and predicts AF development^21^. Furthermore, there was no effect modification by AF status on the relationship between disproportionate LA myopathy and reduced SV in our study, and the associations of disproportionate LA myopathy with poor hemodynamics were independent of AF, suggesting that mechanical dysfunction of the LA uniquely contributes to worse hemodynamics. Furthermore, the 5 unique proteins we identified and validated were associated with disproportionate LA myopathy independent of AF. Finally, we compared the proteomic signatures of disproportionate LA myopathy and AF in both PROMIS-HFpEF and Northwestern cohorts and demonstrated that 10 proteins were uniquely associated with disproportionate LA myopathy, but not AF. The proteomic differences suggest distinct pathophysiology between LA mechanical dysfunction and LA electrical failure (i.e. AF). Taken together, our findings suggest that disproportionate LA myopathy in HFpEF is driven by mechanisms independent of AF alone.

Our study has limitations. While the PROMIS-HFpEF cohort was relatively small, it represents a multinational cohort of prospectively enrolled, well-phenotyped HFpEF. Echocardiographic hemodynamic measures are subject to variability and require confirmation with invasive hemodynamics^22,23^. This investigation was designed to test the hypothesis that there is a subgroup of HFpEF with disproportionate LA myopathy; further investigation is warranted to understand specific clinical thresholds of LA and LV strain that define disproportionate LA myopathy in HFpEF. While the cross-sectional nature of our study prevented the evaluation of longitudinal outcomes and the prognostic utility of disproportionate LA myopathy, LA mechanical dysfunction has been previously associated with adverse clinical outcomes^5^. Given the high rate of AF in those with disproportionate LA myopathy, it is possible that AF is driving the adverse hemodynamic profile in this group, and the cross-sectional nature of our study cannot evaluate the timing of onset of LA myopathy as compared with AF. However, the association of disproportionate LA myopathy with adverse hemodynamics was independent of AF, suggesting that LA mechanical dysfunction contributes uniquely to poor hemodynamics.

Among an international cohort of HFpEF, we identified and characterized individuals with LA myopathy out of proportion to LV myopathy. Despite favorable LV diastolic functional profiles, disproportionate LA myopathy was associated with lower SV and reduced SV reserve, independent of AF. There is a unique proteomic profile that is associated with abnormal LA mechanics in HFpEF independent of AF, highlighted by proteins of cardiomyocyte stretch, collagen homeostasis, and inflammation. In aggregate, disproportionate LA myopathy represents a unique subtype of HFpEF that cannot be fully explained by comorbid AF. HFpEF patients with disproportionate LA myopathy may require distinct therapeutic targeting in order to improve symptoms and prognosis.

## Methods

### Study population

Details regarding the study design for PROMIS-HFpEF have been previously reported^24^. PROMIS-HFpEF enrolled participants with chronic HFpEF between December 2015 and January 2018 across 5 institutions: Karolinska University Hospital (Stockholm, Sweden); Sahlgrenska University Hospital (Gothenburg, Sweden); Turku University Hospital (Turku, Finland); Northwestern Memorial Hospital (Chicago, IL); and National Heart Centre Singapore (Singapore). Detailed inclusion criteria are provided in the Supplemental Methods. For proteomic analyses, we also analyzed data from a validation cohort of HFpEF patients from Northwestern University (n = 117). Detailed information regarding the validation cohort are provided in the Supplemental Methods.

### Echocardiography and coronary flow reserve

Participants underwent 2-dimensional echocardiography, color, spectral, and tissue Doppler imaging, and speckle-tracking (Vivid 7/E9, GE Healthcare, General Electric Corp., Waukesha, WI) as detailed previously^24^. All echocardiographic measurements were performed by a central echocardiography core laboratory (Northwestern University, Chicago, IL). Chamber size, volume, and function were analyzed as outlined by current societal guidelines^22,25,26^. LA and LV volumes were measured by biplane method of discs method in the apical 4- and 2-chamber views. The primary hemodynamic outcome of interest was SV, which was calculated using the LV outflow tract velocity time integral method^27^.

Speckle-tracking echocardiography analysis was performed as previously described^24^. Images were obtained at a frame rate of 50–70 fps and an experienced research sonographer used a software package (GE EchoPAC, GE Healthcare, Wakesha WI, USA) for strain analysis, which was verified by an investigator experienced in echocardiography. Speckle-tracking was not performed in participants with poor image quality (more than 1 myocardial segment unable to be visualized, a missing view, or chamber foreshortening).

The LA endocardial border was traced manually in two views (apical 2- and 4-chamber) for creation of LA longitudinal strain curves. Six segments of the LA were identified by strain software. Segments which did not track appropriately were removed from the analysis and the average of the remaining segments was generated from each view. LA reservoir strain was subsequently calculated by averaging the strain values from both apical views. Given the ventricular cycle was the reference point, all LA strain values were positive. If participants were in AF at the time of echocardiography, speckle-tracking was performed on 3 separate beats and subsequent strain values were averaged. This method has been previously described and validated among those with AF^5^. LV GLS was also calculated through the apical 4-, 3-, and 2-chamber views in similar method. In the case of LV GLS, 6 segments were identified and averaged in each view. RV free wall strain was determined by averaging the 3 RV free wall segments that were identified in the apical 4-chamber RV-focused view. For all strain parameters, the absolute values of strain for each chamber were reported for ease in interpretability (i.e. lower absolute strain values indicate worse mechanical function).

As part of the PROMIS-HFpEF protocol, all participants underwent a standardized passive leg raise (intravascular volume challenge) maneuver. In the supine position, a wedge-shaped pillow was placed under the legs of participants, and they were instructed to keep their legs straight and resting comfortably on the wedge pillow without tensing their leg muscles. Apical 4- and 2-chamber images were obtained during the passive leg raise maneuver, and Doppler (pulsed wave at LV outflow tract and mitral leaflet tips) and tissue Doppler echocardiography was performed.

The protocol for CFR measurement has been previously described^24^. Full details regarding CFR protocol can be found in the Supplemental Methods.

### Proteomic profiling

Plasma proteomic measurements were performed using the Olink Inflammation, Cardiovascular II, and Cardiovascular III panels (Uppsala, Sweden). Full details regarding protein measurement are detailed in the Supplemental Methods. In total, these panels consist of 266 distinct proteins. If protein levels fell below limit of detection in ≥ 50% of the PROMIS-HFpEF cohort, they were excluded from further analysis (n = 18), resulting 248 proteins for analysis.

### Definition of disproportionate left atrial myopathy

We aimed to identify and characterize the cohort of PROMIS-HFpEF by degree of LA myopathy compared with LV myopathy. We defined disproportionate LA myopathy based on the association of LV GLS and LA reservoir strain using linear regression. We chose these 2 indices because LA reservoir strain and LV GLS are 2 of the strongest echocardiographic predictors of adverse cardiovascular outcomes in HFpEF^5,28^. Based on the regression model, continuous studentized residual values were assigned to each participant. Studentized residuals greater than 0 indicate higher than expected LA reservoir strain based on the regression model. Likewise, studentized residuals less than 0 indicate lower than expected levels of LA reservoir strain based on the regression model (i.e., disproportionate LA myopathy).

### Statistical analysis

We used separate univariate linear regression models to identify clinical, laboratory, and echocardiographic variables associated of disproportionate LA myopathy (i.e., lower studentized residuals). Candidate variables were log-transformed when appropriate to maintain homoscedasticity of residuals in the regression models. We additionally evaluated clinical characteristics by dichotomizing studentized residuals into < 0 vs. > 0 groups. We compared the 2 groups with χ^2^ tests for categorical variables and Student’s t tests or Wilcoxon rank sum tests for continuous variables. Multivariable linear regression models evaluated the association of disproportionate LA myopathy (i.e., continuous studentized residual values) with resting SV. Model 1 adjusted for the following variables: age, sex, race, study site, diabetes mellitus, hypertension, BMI, creatinine and average E/e′. Model 2 adjusted for Model 1 covariates plus LV GLS, LA volume, AF, and NT-proBNP. There was no evidence of multi-collinearity of covariates in the fully adjusted models. We assessed the association of studentized residual values with change in SV after passive leg raise with similar linear regression models that further adjusted for resting SV. In sensitivity analysis, we evaluated associations of disproportionate LA myopathy with SV and change in SV after leg raise after further adjustment for NYHA class, 6-min walk distance, and heart rate response to adenosine infusion in addition to Model 2 covariates. Additionally, interaction testing was used to determine whether AF modified the relationship of disproportionate LA myopathy and SV at rest or change in SV after passive leg raise.

Next we evaluated the associations of each of the 248 candidate plasma proteins with disproportionate LA myopathy (i.e., lower residuals) in univariate linear regression analyses. To correct for multiple comparisons, the false discovery rate controlling procedure was used to adjust for multiple hypotheses^29^. We subsequently validated the associations of these plasma proteins in the Northwestern University validation cohort. Of the proteins significantly associated with disproportionate LA myopathy on univariate analysis, we evaluated associations after adjustment for age, sex, and AF in both PROMIS and Northwestern HFpEF cohorts. The protein–protein interactions of top proteins associated with disproportionate LA myopathy in PROMIS and Northwestern HFpEF cohorts were evaluated within the STRING database to further understand the molecular basis of LA myopathy^30^. We performed a similar proteomic analysis to identify proteins associated with AF using logistic regression in PROMIS-HFpEF and subsequently validated these associations in the Northwestern University HFpEF cohort. Two-sided P-values were deemed significant at values < 0.05. All analyses were performed using R version 3.5.1 (R Foundation for Statistical Computing).

### Study approval

This study was approved by the Northwestern University institutional review board and at each of the participating institutions (Karolinska University Hospital; Sahlgrenska University Hospital; Turku University Hospital; and National Heart Centre Singapore). All elements of the research were performed in accordance with relevant guidelines and regulations and informed consent was obtained from all participants. The PROMIS study complies with the Declaration of Helsinki.

## Supplementary Information

Supplementary Information.

## References

[1] Zile MR (2011). Prevalence and significance of alterations in cardiac structure and function in patients with heart failure and a preserved ejection fraction. Circulation.

[2] Melenovsky V (2007). Cardiovascular features of heart failure with preserved ejection fraction versus nonfailing hypertensive left ventricular hypertrophy in the urban Baltimore community: The role of atrial remodeling/dysfunction. J. Am. Coll. Cardiol..

[3] Saraiva RM (2010). Left atrial strain measured by two-dimensional speckle tracking represents a new tool to evaluate left atrial function. J. Am. Soc. Echocardiogr..

[4] Vianna-Pinton R (2009). Two-dimensional speckle-tracking echocardiography of the left atrium: Feasibility and regional contraction and relaxation differences in normal subjects. J. Am. Soc. Echocardiogr..

[5] Freed BH (2016). Prognostic utility and clinical significance of cardiac mechanics in heart failure with preserved ejection fraction: Importance of left atrial strain. Circ. Cardiovasc. Imaging..

[6] Freed BH, Shah SJ (2017). Stepping out of the left ventricle's shadow: Time to focus on the left atrium in heart failure with preserved ejection fraction. Circ. Cardiovasc. Imaging..

[7] Patel RB, Shah SJ (2020). Therapeutic targeting of left atrial myopathy in atrial fibrillation and heart failure with preserved ejection fraction. JAMA Cardiol..

[8] Santos AB (2014). Impaired left atrial function in heart failure with preserved ejection fraction. Eur. J. Heart Fail..

[9] Santos AB (2016). Prognostic relevance of left atrial dysfunction in heart failure with preserved ejection fraction. Circ. Heart Fail..

[10] Telles F (2019). Impaired left atrial strain predicts abnormal exercise haemodynamics in heart failure with preserved ejection fraction. Eur. J. Heart Fail..

[11] von Roeder M (2017). Influence of left atrial function on exercise capacity and left ventricular function in patients with heart failure and preserved ejection fraction. Circ. Cardiovasc. Imaging..

[12] Lundberg A (2019). Left atrial strain improves estimation of filling pressures in heart failure: A simultaneous echocardiographic and invasive haemodynamic study. Clin. Res. Cardiol..

[13] Solomon SD, LA Biering-Sorensen T (2017). Strain when ejection fraction is preserved: A new measure of diastolic function?. JACC Cardiovasc. Imaging.

[14] Ersboll M (2013). The prognostic value of left atrial peak reservoir strain in acute myocardial infarction is dependent on left ventricular longitudinal function and left atrial size. Circ. Cardiovasc. Imaging.

[15] Obokata M (2013). Incremental diagnostic value of la strain with leg lifts in heart failure with preserved ejection fraction. JACC Cardiovasc. Imaging.

[16] Feldman T (2018). Transcatheter interatrial shunt device for the treatment of heart failure with preserved ejection fraction (REDUCE LAP-HF I [Reduce elevated left atrial pressure in patients with heart failure]): A phase 2, randomized, sham-controlled trial. Circulation.

[17] Luchner A (1998). Differential atrial and ventricular expression of myocardial BNP during evolution of heart failure. Am. J. Physiol..

[18] Barallobre-Barreiro J (2016). Glycoproteomics reveals decorin peptides with anti-myostatin activity in human atrial fibrillation. Circulation.

[19] Berntsson J (2019). Increased vascular endothelial growth factor D is associated with atrial fibrillation and ischaemic stroke. Heart.

[20] Bune AJ, Hayman AR, Evans MJ, Cox TM (2001). Mice lacking tartrate-resistant acid phosphatase (Acp 5) have disordered macrophage inflammatory responses and reduced clearance of the pathogen, *Staphylococcus aureus*. Immunology.

[21] Patel RB (2020). Characterization of cardiac mechanics and incident atrial fibrillation in participants of the cardiovascular health study. JCI Insight..

[22] Rudski LG (2010). Guidelines for the echocardiographic assessment of the right heart in adults: A report from the American Society of Echocardiography endorsed by the European Association of Echocardiography, a registered branch of the European Society of Cardiology, and the Canadian Society of Echocardiography. J. Am. Soc. Echocardiogr..

[23] Dubin J, Wallerson DC, Cody RJ, Devereux RB (1990). Comparative accuracy of Doppler echocardiographic methods for clinical stroke volume determination. Am. Heart J..

[24] Shah SJ (2018). Prevalence and correlates of coronary microvascular dysfunction in heart failure with preserved ejection fraction: PROMIS-HFpEF. Eur. Heart J..

[25] Lang RM (2015). Recommendations for cardiac chamber quantification by echocardiography in adults: An update from the American Society of Echocardiography and the European Association of Cardiovascular Imaging. J. Am. Soc. Echocardiogr..

[26] Nagueh SF (2016). Recommendations for the evaluation of left ventricular diastolic function by echocardiography: An update from the American Society of Echocardiography and the European Association of Cardiovascular Imaging. J. Am. Soc. Echocardiogr..

[27] Kirkpatrick JN, Lang RM (2008). Heart failure: Hemodynamic assessment using echocardiography. Curr. Cardiol. Rep..

[28] Shah AM (2015). Prognostic importance of impaired systolic function in heart failure with preserved ejection fraction and the impact of spironolactone. Circulation.

[29] Benjamini Y, Hochberg Y (1995). Controlling the false discovery rate: A practical and powerful approach to multiple testing. J. R. Stat. Soc. Ser. B (Methodol.).

[30] Szklarczyk D (2019). STRING v11: Protein-protein association networks with increased coverage, supporting functional discovery in genome-wide experimental datasets. Nucleic Acids Res..

