# Supplementary material for: Disproportionate left atrial myopathy in heart failure with preserved ejection fraction among participants of the PROMIS-HFpEF study
Source: Sci Rep. 2021 Mar 1;11:4885. doi: 10.1038/s41598-021-84133-9 (PMC7921666; doi:10.1038/s41598-021-84133-9)
Supplement: Supplementary file 1 — Supplementary Information. [file 41598_2021_84133_MOESM1_ESM.docx]

**Disproportionate Left Atrial Myopathy in Heart Failure with Preserved Ejection Fraction Among Participants of the PROMIS-HFpEF Study**

Ravi B. Patel MD MSc, Carolyn S. P. Lam MBBS PhD, Sara Svedlund MD PhD, Antti Saraste MD PhD, Camilla Hage PhD, Ru-San Tan MBBS, Lauren Beussink-Nelson MS, Jasper Tromp MD PhD, Cynthia Sanchez BA, Joyce Njoroge MD, Stanley A. Swat MD, Ulrika Ljung Faxén MD PhD, Maria Lagerstrom Fermer PhD, Ashwin Venkateshvaran PhD, Li-Ming Gan MD PhD, Lars H. Lund MD PhD, Sanjiv J. Shah MD

**SUPPLEMENTAL MATERIAL**

**Supplemental Methods**

**Inclusion Criteria for PROMIS-HFpEF Study**

Inclusion criteria were chronic HFpEF without evidence of significant unrevascularized epicardial coronary artery disease and at least one of the following: (1) elevated N-terminal pro-B-type natriuretic peptide (NT-proBNP) levels; (2) prior hospitalization for HF along with LA chamber enlargement or LV hypertrophy; (3) elevated pulmonary capillary wedge pressures at rest or exercise; or (4) E/e’ ratio >15. Enrolled participants underwent the following testing: history and physical exam, blood and urine sampling, electrocardiography (ECG), comprehensive echocardiography, and Doppler echocardiography with adenosine infusion for measurement of coronary flow reserve (CFR).

**Inclusion Criteria for Northwestern HFpEF validation cohort**

Patients were enrolled in this prospective observational cohort between November 2013 and May 2017 from a HFpEF outpatient clinic. HFpEF was diagnosed by a cardiologist with specific expertise in HFpEF. HFpEF patients were required to have symptomatic HF, LVEF ≥50%, prior history of HF hospitalization, evidence of elevated LV filling pressures (E/e’ > 15 or elevated LV filling pressures on invasive hemodynamic testing), or BNP > 100 pg/ml with evidence of LV hypertrophy or left atrial enlargement based on published guidelines. Patients with dilated, hypertrophic, or infiltrative cardiomyopathy; greater than moderate left-sided valvular disease; congenital heart disease; constrictive pericarditis; or a history of heart transplantation were excluded. There was no patient overlap between the PROMIS-HFpEF and Northwestern HFpEF validation cohorts.

**Coronary Flow Reserve Assessment**

Pulse wave Doppler flow signals before and during adenosine infusion (140 micrograms/min/kg for 5-10 minutes) were acquired at the mid-to-distal portion of the left anterior descending artery in a modified apical 2-chamber view. Mean diastolic flow velocity at baseline and during adenosine infusion was measured by manual tracing and analyzed offline through prespecified software (TOMTEC, Unterschleiβheim, Germany) by a central core laboratory at Sahlgrenska University Hospital (Gothenburg, Sweden). The mean flow velocity of 3 cardiac cycles both at rest and during adenosine was calculated among those in sinus rhythm. Among patients in AF, 10 beats were averaged at both rest and during adenosine infusion. CFR was then calculated as the ratio of average diastolic flow velocity during adenosine to average baseline flow velocity.

**Protein Measurement**

Target-specific antibody pairs linked to DNA strands are used in the Olink Proseek multiplex immunoassay. These paired antibodies linked to DNA creates a polymerase chain reaction upon binding to the target analyte in a proximity-dependent manner. The range of intra-assay coefficient of variation (CV) for protein measurements was 5% to 13%; the range of inter-assay CV for protein measurements was 9% to 39%. The proteins are subsequently expressed as normalized protein units on a log2 scale, where a 1-unit increase represents a doubling of protein concentration. Of 10 proteins (CCL3, CXCL1, OPG, IL-6, uPA, MCP1, SCF, IL-18, FGF-21, and FGF-23) measured on 2 panels, the correlation between panels ranged from 0.87 to 0.97 in both the PROMIS and Northwestern cohorts. The correlation between the NTproBNP Olink assay and a commercially available ELISA immunoassay (Elecsys® NTproBNP assay, Roche Diagnostics GmbH, Mannheim) was 0.89 (P<0.001). In the event that protein levels fell below limit of detection among more than half of the study population, they were excluded (n=18 proteins: IL-20RA, IL-2RB, IL-1α, IL-2, TSLP, FGF-5, IL22RA1, IL-24, IL-13, ARTN, TNF, IL-20, IL-33, IFN-γ, IL4, LIF, NRTN, and IL-5).

**Supplemental Table 1. Characteristics of PROMIS-HFpEF Cohort By Studentized Residual Category.**

| **Characteristic** | **Studentized Residual <0**  (LA>LV myopathy)  N=134 | | | **Studentized Residual >0**  (LA<LV myopathy)  N=107 | | **P value** | |
| --- | --- | --- | --- | --- | --- | --- | --- |
| Age, year | 76.7±7.8 | | | 72.0±9.6 | | <0.001 | |
| Male sex, n (%) | 69 (51.9) | | | 38 (35.5) | | 0.02 | |
| White Race, n (%) | 123 (92.5) | | | 86 (80.4) | | 0.02 | |
| NYHA Class III/IV | 32 (24.1) | | | 29 (27.6) | | 0.64 | |
| **Comorbidities** | | | |  | |  | |
| Hypertension, n (%) | 107 (80.5) | | | 91 (85.0) | | 0.45 | |
| Diabetes, n (%) | 38 (28.6) | | | 25 (23.4) | | 0.45 | |
| Hyperlipidemia, n (%) | 64 (48.1) | | | 64 (59.8) | | 0.09 | |
| Atrial fibrillation, n (%) | 89 (66.4) | | | 10 (9.3) | | <0.001 | |
| ASCVD, n (%) | 57 (42.9) | | | 43 (40.6) | | 0.82 | |
| Chronic kidney disease, n (%) | 78 (58.2) | | | 48 (44.9) | | 0.05 | |
| Pacemaker or ICD, n (%) | 23 (17.3) | | | 18 (16.8) | | 0.99 | |
| **Vital Signs, Physical Characteristics, and Laboratory Data** |  | |  | |  | |  |
| Body mass index, kg/m^2^ | 28.3±5.5 | | | 31.1±10.3 | | 0.007 | |
| Heart rate, bpm | 71.0±14.2 | | | 69.1±12.2 | | 0.27 | |
| SBP, mmHg | 140.2±18.1 | | | 138.3±23.8 | | 0.47 | |
| DBP, mmHg | 78.4±11.0 | | | 73.1±12.0 | | <0.001 | |
| Waist circumference, cm | 100.7±14.7 | | | 102.3±20.9 | | 0.48 | |
| NT-proBNP, pg/mL | | 1280 (800-1883) | | 446 (134-1113) | | <0.001 | |
| Sodium, mEq/L | 140.0±2.8 | | | 139.9±2.9 | | 0.81 | |
| Potassium, mEq/L | 4.3±0.4 | | | 4.2±0.5 | | 0.15 | |
| Creatinine, mg/dL | 1.07 (0.95-1.30) | | | 1.01 (0.83-1.41) | | 0.13 | |
| Glomerular filtration rate, mL/min/1.73 m^2^ | 57.9 (47.6-67.6) | | | 62.4 (42.7-77.4) | | 0.30 | |
| Troponin T, ng/mL | 16.0 (10.0-25.1) | | | 10.3 (10.0-18.0) | | 0.02 | |
| Hemoglobin A1c, % | 40.5 (38.0-49.8) | | | 42.0 (37.0-48.0) | | 0.85 | |
| Urine albumin-to-creatinine ratio, mg/g | 4.70 (1.55-11.66) | | | 1.98 (1.00-7.75) | | 0.03 | |
| **Medications** | | | |  | |  | |
| Loop diuretic, n (%) | 72 (53.7) | | | 58 (54.2) | | 0.99 | |
| Thiazide diuretic, n (%) | 11 (8.2) | | | 15 (14.0) | | 0.22 | |
| Mineralocorticoid receptor antagonist, n (%) | 35 (26.1) | | | 34 (31.8) | | 0.41 | |
| ACE inhibitor, n (%) | 41 (30.6) | | | 30 (28.0) | | 0.77 | |
| ARB, n (%) | 56 (41.8) | | | 46 (43.0) | | 0.96 | |

Continuous variables are listed as mean±SD or median (interquartile range) depending on distribution.

ACE = angiotensin converting enzyme; ARB = angiotensin receptor blocker; ASCVD = atherosclerotic vascular disease; DBP = diastolic blood pressure; GFR = glomerular filtrate rate; ICD = implantable cardioverter-defibrillator; LA = left atrial; LV = left ventricular; NT-proBNP = N-terminal pro-B-type natriuretic peptide; NYHA = New York Heart Association; SBP = systolic blood pressure; UACR = urinary albumin-to-creatinine ratio.

**Supplemental Table 2.** **Sensitivity Analysis:** **Association of Disproportionate Left Atrial Myopathy with Stroke Volume and Stroke Volume Reserve (after passive leg raise maneuver).**

|  | | **β-coefficient per 1-unit decrease in studentized residual (95% CI)** | **P value** |
| --- | --- | --- | --- |
| **Resting SV** | | |  |
| Model 1† | | -5.2 (-8.0, -2.4) | <0.001 |
| Model 2‡ | | -7.3 (-10.9, -3.7) | <0.001 |
| **Change in SV after leg raise§** |  | |  |
| Model 1† | | -1.5 (-3.0, -0.04) | 0.04 |
| Model 2‡ | | -2.2 (-4.3, -0.1) | 0.04 |

BMI = body mass index; GLS= global longitudinal strain; LA = left atrial; LAVI = left atrial volume; LV = left ventricular; NT-proBNP = N-terminal pro-B-type natriuretic peptide; SV = stroke volume.

† Adjusted for enrollment site, age, sex, race, BMI, hypertension, diabetes, creatinine, and average E/e’

‡ Adjusted for Model 1 covariates plus LV GLS, LAV, NT-proBNP, AF, NYHA class, 6MWD, and heart rate response to adenosine

§ All models adjusted for resting SV

**Supplemental Table 3. Plasma Proteins Associated with Disproportionate LA Myopathy on Univariate Linear Regression in PROMIS Cohort and Northwestern HFpEF (Validation) Cohort.**

|  | **PROMIS Cohort (n=241)** | | | | **Northwestern HFpEF Cohort (n=117)** | | | |
| --- | --- | --- | --- | --- | --- | --- | --- | --- |
| **Protein** | **β-coefficient** | **SE** | **P value*** | **Model R^2^** | **β-coefficient** | **SE** | **P value** | **Model R^2^** |
| NTproBNP† | 0.30 | 0.04 | 3.6x10^-8^ | 0.18 | 0.28 | 0.06 | 7.78 x10^-6^ | 0.19 |
| BNP† | 0.26 | 0.05 | 1.04 x10^-5^ | 0.13 | 0.23 | 0.05 | 3.22 x10^-5^ | 0.16 |
| HGF | 0.73 | 0.13 | 1.26 x10^-5^ | 0.12 | -0.02 | 0.11 | 0.86 | 0.0003 |
| RAGE† | 0.65 | 0.13 | 9.42 x10^-5^ | 0.10 | 0.44 | 0.17 | 0.01 | 0.07 |
| PRELP† | 0.95 | 0.22 | 0.001 | 0.08 | 1.38 | 0.37 | 0.0003 | 0.13 |
| PSPD | 0.37 | 0.09 | 0.001 | 0.08 | 0.18 | 0.12 | 0.12 | 0.03 |
| TRAP† | -0.62 | 0.15 | 0.002 | 0.08 | -0.45 | 0.18 | 0.01 | 0.06 |
| IGFBP7† | 0.50 | 0.12 | 0.002 | 0.07 | 0.40 | 0.16 | 0.01 | 0.06 |
| MMP2 | 0.56 | 0.14 | 0.002 | 0.07 | 0.30 | 0.16 | 0.15 | 0.02 |
| Notch3† | 0.60 | 0.16 | 0.004 | 0.07 | 0.38 | 0.16 | 0.02 | 0.05 |
| TFPI | -0.69 | 0.19 | 0.01 | 0.06 | -0.33 | 0.16 | 0.08 | 0.03 |
| ACE2† | 0.32 | 0.09 | 0.01 | 0.06 | 0.32 | 0.16 | 0.02 | 0.05 |
| SPON1 | 0.85 | 0.24 | 0.01 | 0.05 | 0.09 | 0.16 | 0.51 | 0.004 |
| FGF23† | 0.18 | 0.05 | 0.02 | 0.05 | 0.16 | 0.16 | 0.03 | 0.05 |
| CXCL10 | 0.22 | 0.07 | 0.03 | 0.05 | 0.004 | 0.16 | 0.97 | 1.32 x10^-5^ |
| BetaNGF | 0.48 | 0.15 | 0.03 | 0.04 | 0.28 | 0.23 | 0.23 | 0.02 |
| CCL20 | 0.19 | 0.06 | 0.04 | 0.04 | 0.005 | 0.08 | 0.95 | 4.63 x10^-5^ |
| DCN† | 0.45 | 0.15 | 0.04 | 0.04 | 0.68 | 0.23 | 0.004 | 0.08 |
| VEGFD† | 0.29 | 0.10 | 0.04 | 0.04 | 0.66 | 0.19 | 0.0008 | 0.11 |
| LIFR† | 0.65 | 0.22 | 0.04 | 0.04 | 0.53 | 0.23 | 0.02 | 0.05 |
| OPG† | 0.43 | 0.14 | 0.04 | 0.04 | 0.38 | 0.17 | 0.02 | 0.05 |

*False discovery rate-adjusted values; †Proteins significantly associated with LA reservoir strain in both PROMIS-HFpEF and Northwestern HFpEF cohort

|  | **Disproportionate LA Myopathy** | | | | | | **AF** | | | | | | |
| --- | --- | --- | --- | --- | --- | --- | --- | --- | --- | --- | --- | --- | --- |
|  | **PROMIS** | | | **Northwestern** | | |  | **PROMIS** | | | **Northwestern** | | |
| **Protein** | **β-coefficient** | **SE** | **P value*** | **β-coefficient** | **SE** | **P value** | **Protein** | **B coefficient** | **SE** | **P value** | **B coefficient** | **SE** | **P value** |
| NTproBNP | 0.30 | 0.04 | 3.6x10^-8^ | 0.28 | 0.06 | 7.78 x10^-6^ | NTproBNP | 0.61 | 0.12 | 0.0002 | 0.43 | 0.19 | 0.02 |
| BNP | 0.26 | 0.05 | 1.04 x10^-5^ | 0.23 | 0.05 | 3.22 x10^-5^ | BNP | 0.42 | 0.11 | 0.006 | 0.42 | 0.17 | 0.01 |
| RAGE† | 0.65 | 0.13 | 9.42 x10^-5^ | 0.44 | 0.17 | 0.01 | ACE2 | 0.83 | 0.21 | 0.006 | 0.81 | 0.34 | 0.02 |
| PRELP† | 0.95 | 0.22 | 0.001 | 1.38 | 0.37 | 0.0003 |  |  |  |  |  |  |  |
| TRAP† | -0.62 | 0.15 | 0.002 | -0.45 | 0.18 | 0.01 |  |  |  |  |  |  |  |
| IGFBP7† | 0.50 | 0.12 | 0.002 | 0.40 | 0.16 | 0.01 |  |  |  |  |  |  |  |
| Notch3† | 0.60 | 0.16 | 0.004 | 0.38 | 0.16 | 0.02 |  |  |  |  |  |  |  |
| ACE2 | 0.32 | 0.09 | 0.01 | 0.32 | 0.16 | 0.02 |  |  |  |  |  |  |  |
| FGF23† | 0.18 | 0.05 | 0.02 | 0.16 | 0.16 | 0.03 |  |  |  |  |  |  |  |
| DCN† | 0.45 | 0.15 | 0.04 | 0.68 | 0.23 | 0.004 |  |  |  |  |  |  |  |
| VEGFD† | 0.29 | 0.10 | 0.04 | 0.66 | 0.19 | 0.0008 |  |  |  |  |  |  |  |
| LIFR† | 0.65 | 0.22 | 0.04 | 0.53 | 0.23 | 0.02 |  |  |  |  |  |  |  |
| OPG† | 0.43 | 0.14 | 0.04 | 0.38 | 0.17 | 0.02 |  |  |  |  |  |  |  |

**Supplemental Table 4. Proteins associated with Disproportionate LA myopathy and AF in both derivation and validation cohorts.**

† Proteins associated with disproportionate LA myopathy, but not AF, in both derivation and validation cohorts

**Supplemental Figure 1. Association of coronary flow reserve with disproportionate LA myopathy.** Shown is the relationship between coronary flow reserve and the residual values of the regression model between LV GLS and LA reservoir strain (lower residual values indicate disproportionate LA myopathy). The red line represents the linear regression model between the 2 variables. Lower coronary flow reserve was associated with disproportionate LA myopathy.

**
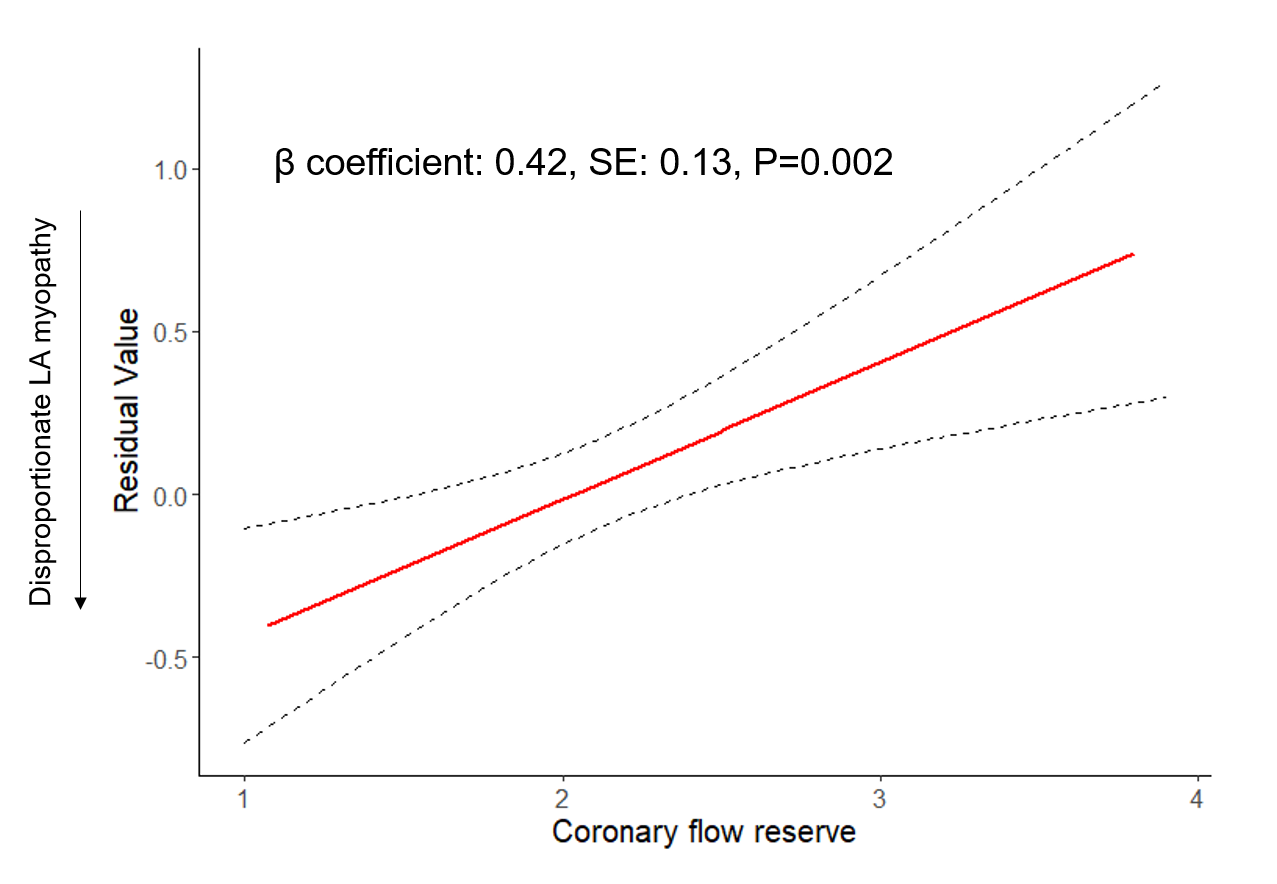
**

**Supplemental Figure 2. Waterfall plot of change in stroke volume after intravascular volume challenge.** Each bar represents a participant in the PROMIS-HFpEF cohort.


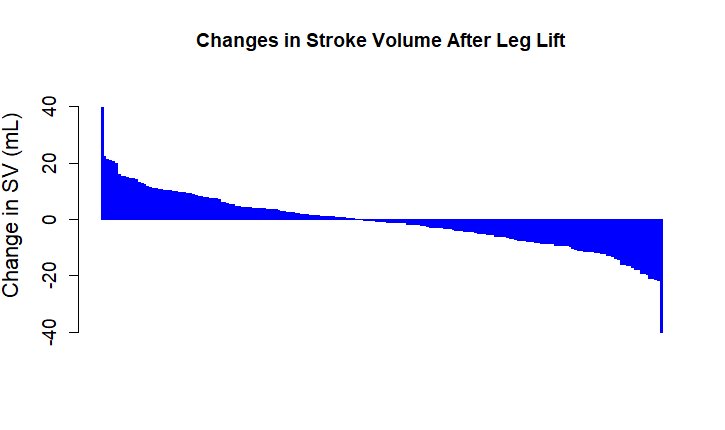


**Supplemental Figure 3. Association of Plasma Proteins with Atrial Fibrillation in PROMIS-HFpEF.** Volcano plot shows proteins positively and negatively associated with AF. The y-axis represents false-discovery rate adjusted P-values. There were 11 proteins associated with AF (red) after adjustment for multiple comparisons.

**
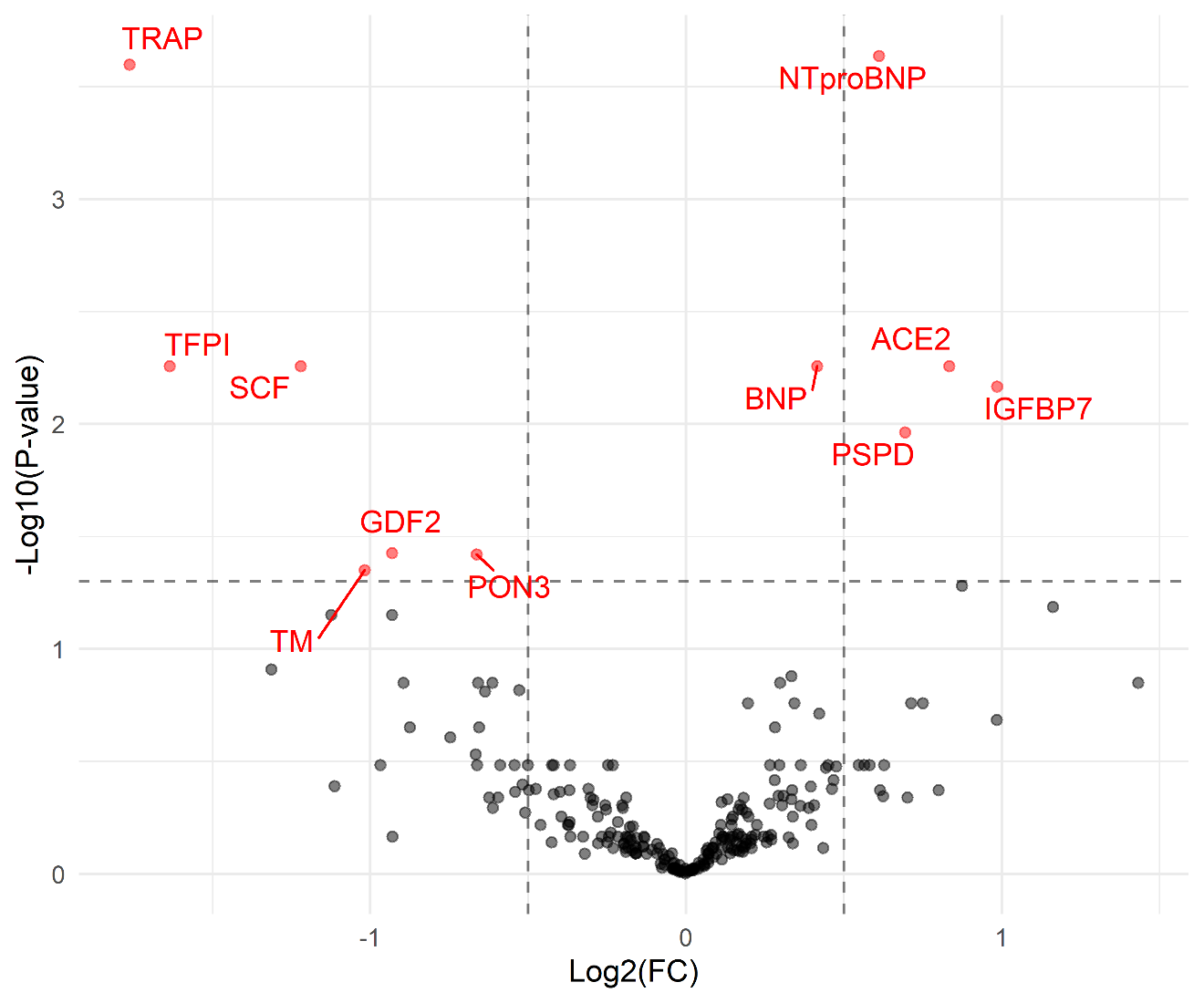
**

**Supplemental Figure 4. Comparison of Validated Proteins Associated with Disproportionate LA Myopathy and AF.** Shown is a Venn Diagram of proteins associated with either disproportionate LA myopathy, AF, or both in both PROMIS-HFpEF and Northwestern HFpEF (validation) cohorts. Proteins listed were significantly associated with either phenotype after adjustment for multiple comparisons (false-discovery rate method).

**
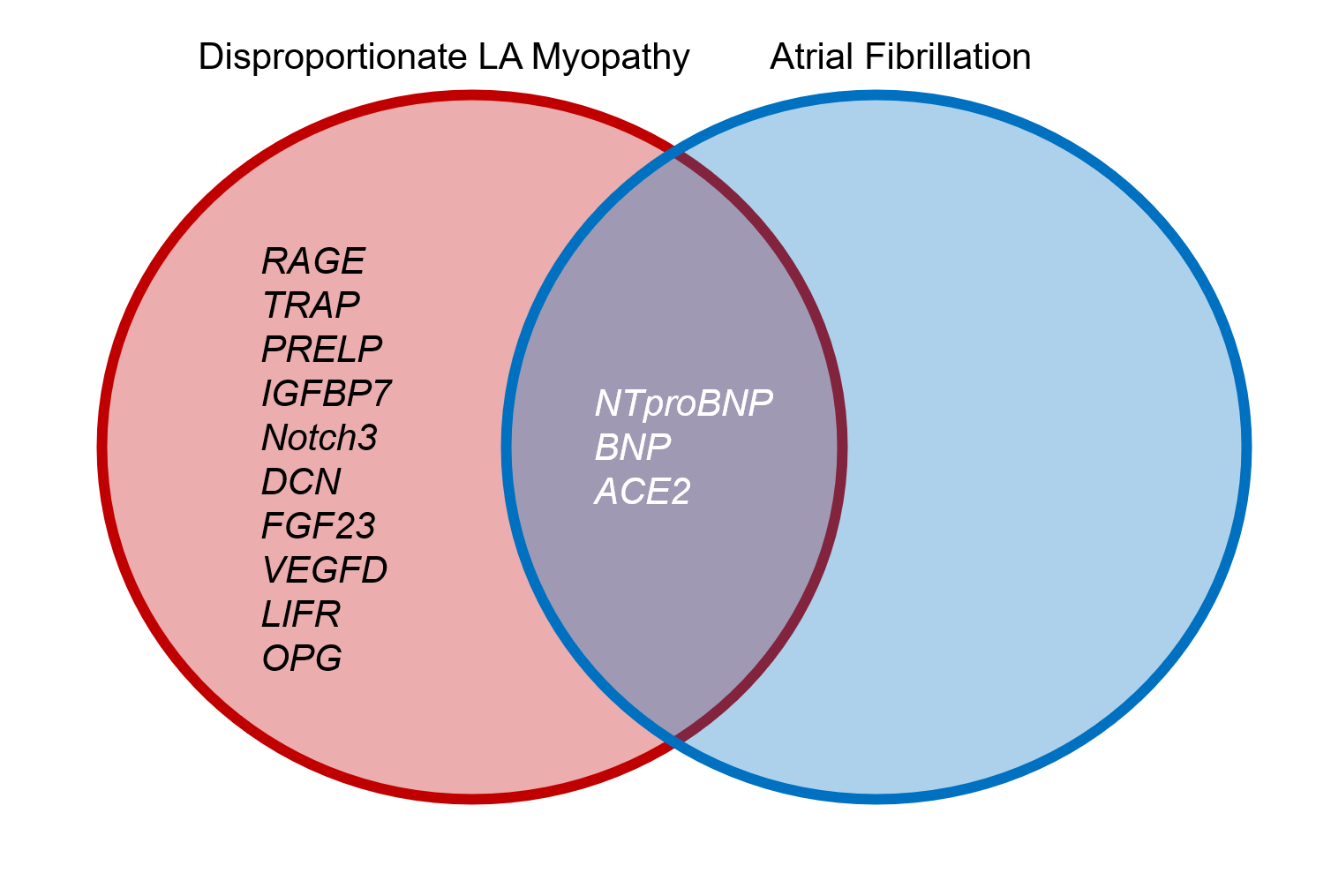
**

**Supplemental Figure 5**. Protein-Protein Interactions for Decorin.^1^*


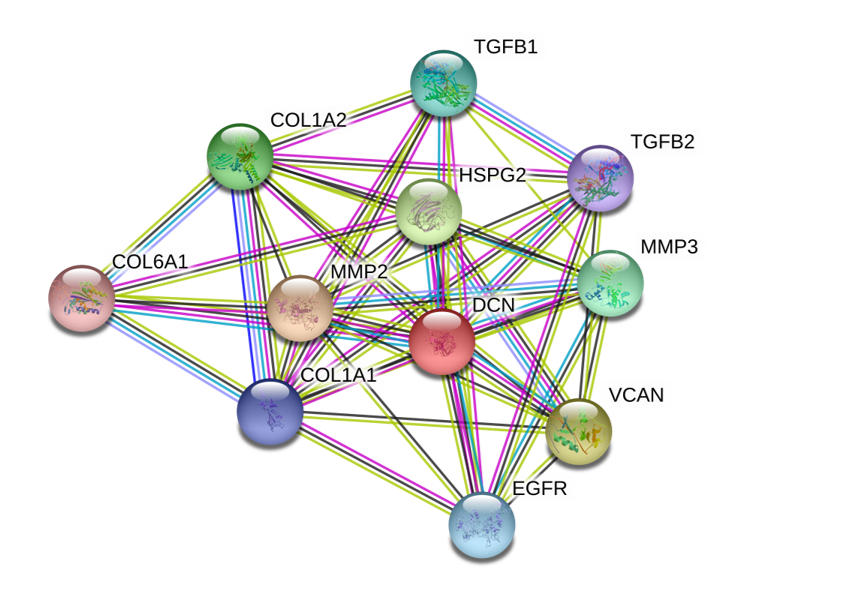


| Node 1 | Node 2 | Experimentally Determined Association | Database | Automated Text Mining | Combined Score |
| --- | --- | --- | --- | --- | --- |
| COL1A2 | COL1A1 | 0.45 | 0.9 | 0.948 | 0.999 |
| COL6A1 | COL1A1 | 0 | 0.9 | 0.693 | 0.994 |
| MMP2 | DCN | 0.38 | 0.9 | 0.58 | 0.993 |
| COL6A1 | COL1A2 | 0 | 0.9 | 0.661 | 0.989 |
| VCAN | DCN | 0.09 | 0.9 | 0.852 | 0.985 |
| HSPG2 | DCN | 0.09 | 0.9 | 0.742 | 0.975 |
| COL1A2 | DCN | 0.41 | 0 | 0.556 | 0.974 |
| MMP3 | DCN | 0.38 | 0.9 | 0.581 | 0.973 |
| COL1A1 | MMP2 | 0.38 | 0 | 0.6 | 0.97 |
| TGFB1 | DCN | 0.39 | 0.9 | 0.542 | 0.969 |
| EGFR | DCN | 0.4 | 0.8 | 0.746 | 0.968 |
| COL1A1 | DCN | 0.41 | 0 | 0.61 | 0.966 |
| TGFB2 | DCN | 0.39 | 0.9 | 0.4 | 0.961 |
| COL6A1 | DCN | 0.41 | 0.36 | 0.453 | 0.96 |
| MMP3 | EGFR | 0 | 0.9 | 0.543 | 0.954 |
| COL1A2 | MMP2 | 0 | 0 | 0.721 | 0.949 |
| HSPG2 | VCAN | 0.19 | 0.9 | 0.709 | 0.943 |
| HSPG2 | MMP3 | 0 | 0.9 | 0.355 | 0.934 |
| TGFB2 | TGFB1 | 0.32 | 0.9 | 0.793 | 0.934 |
| MMP3 | MMP2 | 0 | 0.9 | 0.892 | 0.923 |
| COL6A1 | MMP2 | 0.14 | 0 | 0.334 | 0.864 |
| TGFB1 | MMP2 | 0.52 | 0 | 0.676 | 0.842 |
| COL1A1 | TGFB1 | 0.3 | 0 | 0.651 | 0.75 |
| EGFR | MMP2 | 0 | 0 | 0.673 | 0.697 |
| TGFB2 | MMP2 | 0 | 0 | 0.654 | 0.676 |
| HSPG2 | EGFR | 0.33 | 0 | 0.433 | 0.651 |
| HSPG2 | COL1A1 | 0.16 | 0 | 0.473 | 0.643 |
| MMP3 | COL1A1 | 0 | 0 | 0.567 | 0.621 |
| TGFB2 | VCAN | 0 | 0 | 0.608 | 0.616 |
| EGFR | VCAN | 0 | 0 | 0.609 | 0.612 |
| COL1A2 | TGFB1 | 0.08 | 0 | 0.559 | 0.585 |
| MMP3 | COL1A2 | 0 | 0 | 0.522 | 0.574 |
| VCAN | COL1A1 | 0 | 0 | 0.519 | 0.564 |
| HSPG2 | COL6A1 | 0.16 | 0 | 0.41 | 0.562 |
| EGFR | TGFB1 | 0.15 | 0 | 0.5 | 0.556 |
| COL1A2 | VCAN | 0 | 0 | 0.473 | 0.547 |
| TGFB2 | EGFR | 0.08 | 0 | 0.415 | 0.541 |
| HSPG2 | MMP2 | 0 | 0 | 0.447 | 0.541 |
| TGFB2 | COL1A1 | 0.08 | 0 | 0.48 | 0.535 |
| MMP3 | TGFB1 | 0 | 0 | 0.512 | 0.512 |
| VCAN | MMP2 | 0 | 0 | 0.476 | 0.506 |
| TGFB2 | COL1A2 | 0.08 | 0 | 0.448 | 0.5 |
| MMP3 | VCAN | 0 | 0 | 0.487 | 0.497 |
| EGFR | COL1A1 | 0.1 | 0 | 0.339 | 0.464 |
| HSPG2 | COL1A2 | 0.16 | 0 | 0.266 | 0.435 |

DCN = decorin

*Protein-Protein Interactions are reported from the STRING database (<https://version11.string-db.org/cgi/input.pl?sessionId=wGPwQISZgRcC&input_page_show_search=on>)

**Supplemental Figure 6**. Protein-Protein Interactions for PRELP.^1^*


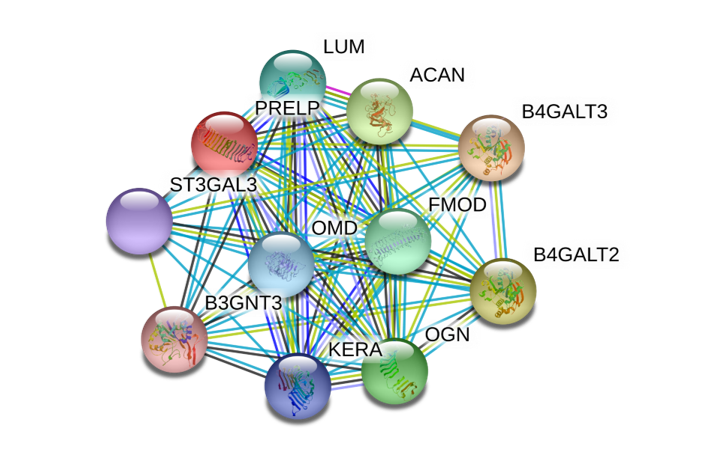


| Node 1 | Node 2 | Experimentally Determined Association | Database | Automated Text Mining | Combined Score |
| --- | --- | --- | --- | --- | --- |
| ACAN | FMOD | 0 | 0.9 | 0.814 | 0.981 |
| ACAN | LUM | 0.379 | 0.9 | 0.669 | 0.978 |
| B4GALT3 | PRELP | 0 | 0.9 | 0.747 | 0.973 |
| PRELP | B4GALT2 | 0 | 0.9 | 0.746 | 0.973 |
| B4GALT2 | ST3GAL3 | 0 | 0.9 | 0.672 | 0.966 |
| ACAN | OMD | 0 | 0.9 | 0.67 | 0.965 |
| OMD | OGN | 0 | 0.9 | 0.854 | 0.964 |
| ACAN | PRELP | 0 | 0.9 | 0.551 | 0.954 |
| ACAN | KERA | 0 | 0.9 | 0.448 | 0.943 |
| ACAN | OGN | 0 | 0.9 | 0.442 | 0.943 |
| LUM | OGN | 0 | 0.9 | 0.819 | 0.942 |
| FMOD | LUM | 0 | 0.9 | 0.915 | 0.938 |
| PRELP | OGN | 0 | 0.9 | 0.806 | 0.935 |
| FMOD | OGN | 0 | 0.9 | 0.769 | 0.935 |
| B4GALT3 | ST3GAL3 | 0 | 0.9 | 0.364 | 0.933 |
| KERA | OGN | 0 | 0.9 | 0.856 | 0.933 |
| B3GNT3 | B4GALT2 | 0 | 0.9 | 0.341 | 0.931 |
| B4GALT3 | B3GNT3 | 0 | 0.9 | 0.338 | 0.931 |
| FMOD | PRELP | 0 | 0.9 | 0.901 | 0.926 |
| KERA | LUM | 0 | 0.9 | 0.935 | 0.917 |
| OMD | FMOD | 0 | 0.9 | 0.742 | 0.917 |
| PRELP | LUM | 0 | 0.9 | 0.818 | 0.916 |
| FMOD | KERA | 0 | 0.9 | 0.764 | 0.916 |
| OMD | PRELP | 0 | 0.9 | 0.776 | 0.914 |
| OMD | LUM | 0 | 0.9 | 0.731 | 0.911 |
| B4GALT2 | OGN | 0 | 0.9 | 0.128 | 0.911 |
| B4GALT3 | LUM | 0 | 0.9 | 0.143 | 0.91 |
| B4GALT2 | LUM | 0 | 0.9 | 0.126 | 0.908 |
| B4GALT3 | OGN | 0 | 0.9 | 0.118 | 0.908 |
| PRELP | KERA | 0 | 0.9 | 0.787 | 0.908 |
| FMOD | B4GALT2 | 0 | 0.9 | 0.094 | 0.907 |
| OMD | KERA | 0 | 0.9 | 0.666 | 0.907 |
| B4GALT3 | FMOD | 0 | 0.9 | 0.093 | 0.905 |
| PRELP | ST3GAL3 | 0 | 0.9 | 0 | 0.902 |
| B3GNT3 | OGN | 0 | 0.9 | 0 | 0.901 |
| PRELP | B3GNT3 | 0 | 0.9 | 0 | 0.901 |
| B3GNT3 | KERA | 0 | 0.9 | 0 | 0.901 |
| FMOD | B3GNT3 | 0 | 0.9 | 0 | 0.901 |
| OMD | B3GNT3 | 0 | 0.9 | 0.041 | 0.901 |
| B3GNT3 | LUM | 0 | 0.9 | 0 | 0.901 |
| B4GALT3 | KERA | 0 | 0.9 | 0.046 | 0.9 |
| KERA | ST3GAL3 | 0 | 0.9 | 0 | 0.9 |
| ACAN | B3GNT3 | 0 | 0.9 | 0 | 0.9 |
| OMD | B4GALT2 | 0 | 0.9 | 0 | 0.9 |
| B4GALT3 | ACAN | 0 | 0.9 | 0 | 0.9 |
| B4GALT2 | KERA | 0 | 0.9 | 0 | 0.9 |
| ACAN | B4GALT2 | 0 | 0.9 | 0 | 0.9 |
| LUM | ST3GAL3 | 0 | 0.9 | 0 | 0.9 |
| FMOD | ST3GAL3 | 0 | 0.9 | 0 | 0.9 |
| OMD | ST3GAL3 | 0 | 0.9 | 0 | 0.9 |
| ST3GAL3 | OGN | 0 | 0.9 | 0 | 0.9 |
| ACAN | ST3GAL3 | 0 | 0.9 | 0 | 0.9 |
| B4GALT3 | OMD | 0 | 0.9 | 0 | 0.9 |
| B4GALT3 | B4GALT2 | 0 | 0.8 | 0.887 | 0.816 |
| B3GNT3 | ST3GAL3 | 0 | 0 | 0.438 | 0.438 |

PRELP= proline-arginine-rich end leucine-rich repeat protein

*Protein-Protein Interactions are reported from the STRING database (<https://version11.string-db.org/cgi/input.pl?sessionId=wGPwQISZgRcC&input_page_show_search=on>)

**Supplemental Figure 7**. Protein-Protein Interactions for VEGFD.^1^*


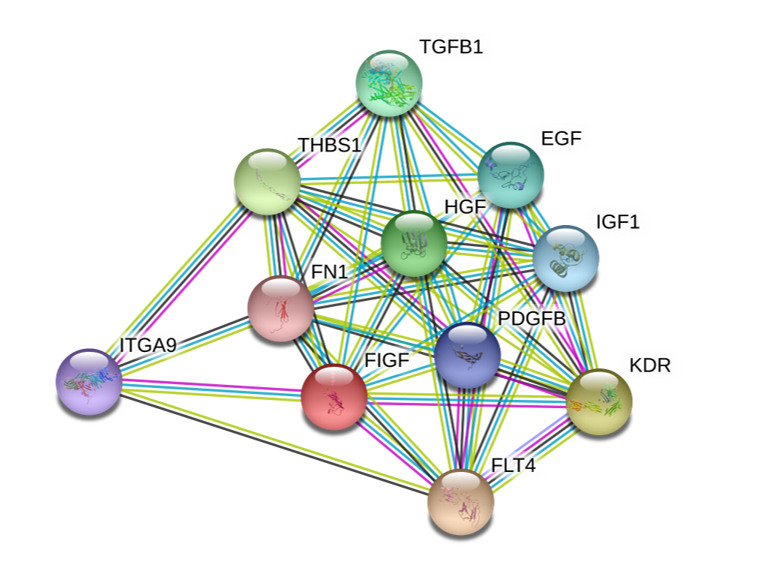


| Node 1 | Node 2 | Experimentally Determined Association | Database | Automated Text Mining | Combined Score |
| --- | --- | --- | --- | --- | --- |
| FIGF | FLT4 | 0.438 | 0.9 | 0.958 | 0.997 |
| FIGF | KDR | 0.472 | 0.9 | 0.919 | 0.995 |
| IGF1 | EGF | 0 | 0.9 | 0.926 | 0.992 |
| FN1 | EGF | 0 | 0.9 | 0.888 | 0.988 |
| FN1 | TGFB1 | 0 | 0.9 | 0.859 | 0.985 |
| EGF | HGF | 0 | 0.9 | 0.854 | 0.984 |
| FN1 | HGF | 0.518 | 0.9 | 0.69 | 0.984 |
| FN1 | THBS1 | 0.379 | 0.9 | 0.692 | 0.982 |
| THBS1 | TGFB1 | 0.379 | 0.9 | 0.722 | 0.981 |
| IGF1 | HGF | 0 | 0.9 | 0.785 | 0.978 |
| THBS1 | HGF | 0 | 0.9 | 0.744 | 0.973 |
| EGF | THBS1 | 0 | 0.9 | 0.731 | 0.972 |
| HGF | TGFB1 | 0 | 0.9 | 0.712 | 0.97 |
| IGF1 | TGFB1 | 0 | 0.9 | 0.705 | 0.969 |
| FN1 | IGF1 | 0 | 0.9 | 0.69 | 0.968 |
| EGF | TGFB1 | 0 | 0.9 | 0.67 | 0.965 |
| FIGF | THBS1 | 0 | 0.9 | 0.625 | 0.961 |
| PDGFB | THBS1 | 0.379 | 0.9 | 0.399 | 0.959 |
| PDGFB | EGF | 0 | 0.9 | 0.58 | 0.957 |
| KDR | FLT4 | 0.472 | 0.9 | 0.954 | 0.952 |
| FIGF | HGF | 0 | 0.9 | 0.531 | 0.952 |
| PDGFB | TGFB1 | 0 | 0.9 | 0.529 | 0.95 |
| PDGFB | IGF1 | 0 | 0.9 | 0.529 | 0.95 |
| FIGF | TGFB1 | 0 | 0.9 | 0.514 | 0.949 |
| FN1 | PDGFB | 0 | 0.9 | 0.514 | 0.949 |
| PDGFB | HGF | 0 | 0.9 | 0.488 | 0.946 |
| FIGF | EGF | 0 | 0.9 | 0.491 | 0.946 |
| IGF1 | FIGF | 0 | 0.9 | 0.479 | 0.945 |
| FN1 | FLT4 | 0 | 0.9 | 0.445 | 0.943 |
| IGF1 | THBS1 | 0 | 0.9 | 0.445 | 0.943 |
| PDGFB | FIGF | 0 | 0.9 | 0.448 | 0.942 |
| FIGF | ITGA9 | 0.379 | 0.9 | 0.146 | 0.942 |
| FN1 | ITGA9 | 0 | 0.9 | 0.412 | 0.939 |
| FN1 | FIGF | 0 | 0.9 | 0.377 | 0.936 |
| PDGFB | KDR | 0.393 | 0.6 | 0.746 | 0.935 |
| PDGFB | FLT4 | 0.393 | 0.6 | 0.66 | 0.912 |
| EGF | KDR | 0.062 | 0.6 | 0.71 | 0.881 |
| KDR | HGF | 0 | 0.6 | 0.676 | 0.867 |
| IGF1 | KDR | 0 | 0.6 | 0.625 | 0.846 |
| ITGA9 | THBS1 | 0.13 | 0.6 | 0.508 | 0.818 |
| EGF | FLT4 | 0.062 | 0.6 | 0.511 | 0.8 |
| FLT4 | HGF | 0 | 0.6 | 0.511 | 0.8 |
| IGF1 | FLT4 | 0 | 0.6 | 0.399 | 0.754 |
| FN1 | KDR | 0 | 0 | 0.687 | 0.701 |
| KDR | THBS1 | 0 | 0 | 0.602 | 0.602 |
| KDR | TGFB1 | 0.078 | 0 | 0.411 | 0.446 |
| ITGA9 | FLT4 | 0 | 0 | 0.391 | 0.419 |

FIGF = vascular endothelial growth factor D

*Protein-Protein Interactions are reported from the STRING database (<https://version11.string-db.org/cgi/input.pl?sessionId=wGPwQISZgRcC&input_page_show_search=on>)

**Supplemental Figure 8**. Protein-Protein Interactions for TRAP.^1^*


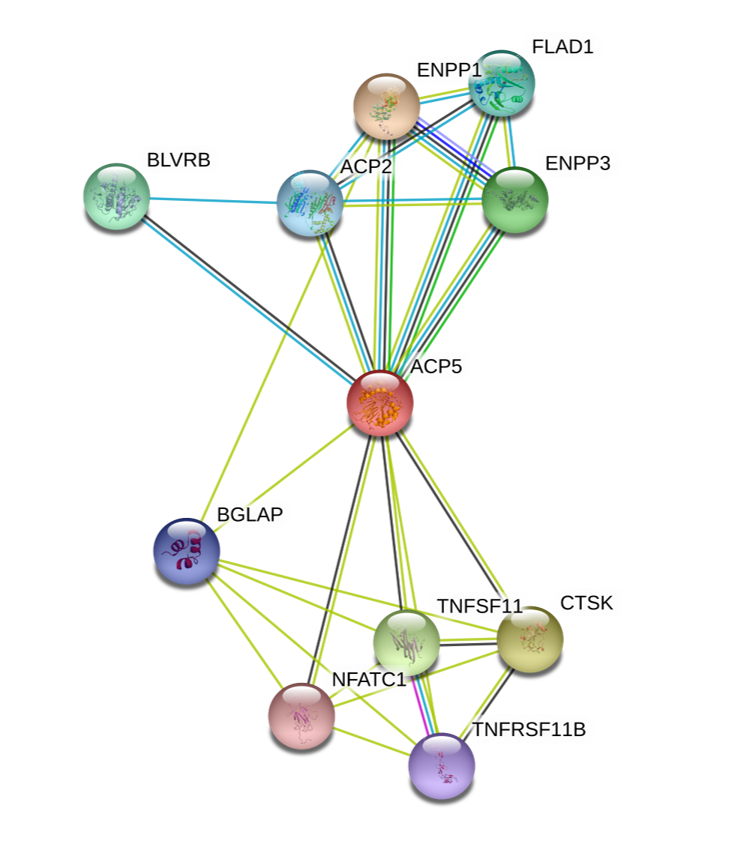


| Node 1 | Node 2 | Experimentally Determined Association | Database | Automated Text Mining | Combined Score |
| --- | --- | --- | --- | --- | --- |
| TNFSF11 | TNFRSF11B | 0.94 | 0.9 | 0.96 | 0.999 |
| TNFSF11 | CTSK | 0 | 0 | 0.961 | 0.962 |
| NFATC1 | TNFSF11 | 0 | 0 | 0.958 | 0.959 |
| ENPP1 | ACP2 | 0 | 0.9 | 0.342 | 0.931 |
| ACP5 | ENPP1 | 0 | 0.9 | 0.287 | 0.929 |
| ACP5 | CTSK | 0 | 0 | 0.921 | 0.928 |
| NFATC1 | CTSK | 0 | 0 | 0.924 | 0.924 |
| ENPP3 | ACP2 | 0 | 0.9 | 0.253 | 0.922 |
| ACP5 | TNFSF11 | 0 | 0 | 0.921 | 0.922 |
| ACP5 | ENPP3 | 0 | 0.9 | 0.108 | 0.912 |
| ACP5 | BLVRB | 0 | 0.9 | 0 | 0.904 |
| ACP5 | FLAD1 | 0 | 0.9 | 0.05 | 0.903 |
| FLAD1 | ACP2 | 0 | 0.9 | 0 | 0.902 |
| BLVRB | ACP2 | 0 | 0.9 | 0 | 0.9 |
| BGLAP | TNFRSF11B | 0 | 0 | 0.892 | 0.892 |
| ACP5 | ACP2 | 0 | 0.8 | 0.445 | 0.887 |
| ACP5 | BGLAP | 0 | 0 | 0.87 | 0.87 |
| TNFSF11 | BGLAP | 0 | 0 | 0.866 | 0.866 |
| ACP5 | TNFRSF11B | 0 | 0 | 0.86 | 0.86 |
| ACP5 | NFATC1 | 0 | 0 | 0.836 | 0.841 |
| ENPP3 | FLAD1 | 0 | 0.8 | 0.19 | 0.831 |
| ENPP1 | FLAD1 | 0 | 0.8 | 0.135 | 0.819 |
| ENPP3 | ENPP1 | 0 | 0.8 | 0.731 | 0.815 |
| TNFRSF11B | CTSK | 0 | 0 | 0.769 | 0.774 |
| BGLAP | CTSK | 0 | 0 | 0.71 | 0.71 |
| NFATC1 | TNFRSF11B | 0 | 0 | 0.664 | 0.664 |
| NFATC1 | BGLAP | 0 | 0 | 0.558 | 0.558 |
| BGLAP | ENPP1 | 0 | 0 | 0.441 | 0.441 |

ACP5 = tartrate resistant acid phosphatase-5

*Protein-Protein Interactions are reported from the STRING database (<https://version11.string-db.org/cgi/input.pl?sessionId=wGPwQISZgRcC&input_page_show_search=on>)

**Supplemental Figure 9**. Protein-Protein Interactions for BNP.^1^*


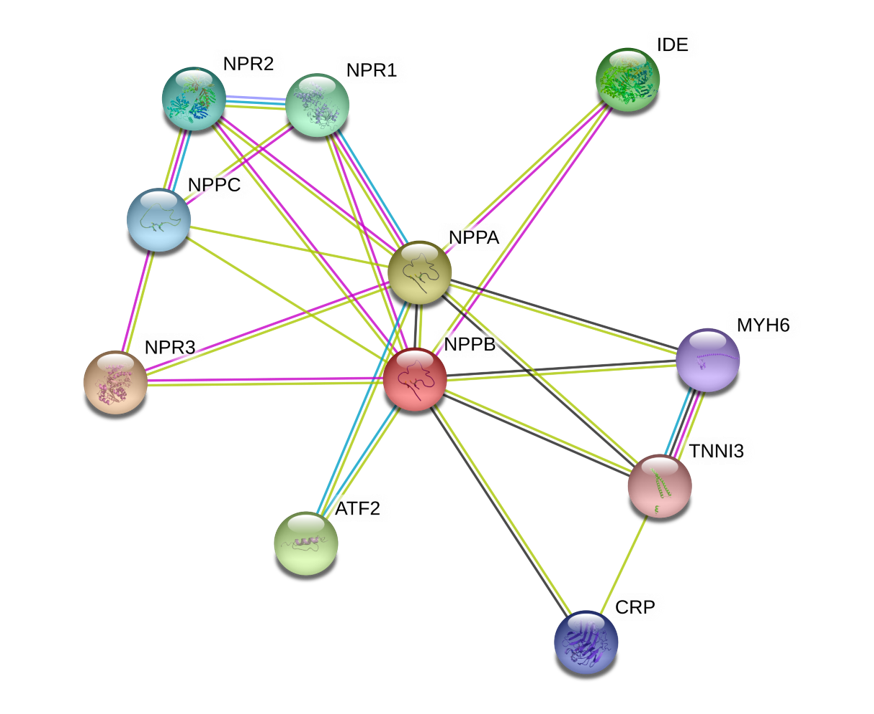


| Node 1 | Node 2 | Experimentally Determined Association | Database | Automated Text Mining | Combined Score |
| --- | --- | --- | --- | --- | --- |
| NPPA | NPR1 | 0.831 | 0.9 | 0.88 | 0.997 |
| NPPC | NPR2 | 0.576 | 0.9 | 0.953 | 0.997 |
| MYH6 | TNNI3 | 0.157 | 0.9 | 0.824 | 0.987 |
| NPPA | NPR3 | 0.886 | 0 | 0.874 | 0.985 |
| NPPC | NPR3 | 0.72 | 0 | 0.897 | 0.97 |
| NPPB | NPR3 | 0.87 | 0 | 0.738 | 0.964 |
| NPPC | NPR1 | 0.547 | 0 | 0.891 | 0.948 |
| NPPA | NPR2 | 0.813 | 0 | 0.73 | 0.947 |
| NPPA | NPPB | 0 | 0 | 0.923 | 0.932 |
| NPPA | ATF2 | 0 | 0.9 | 0.095 | 0.905 |
| NPPB | ATF2 | 0 | 0.9 | 0.086 | 0.904 |
| NPPA | IDE | 0.83 | 0 | 0.203 | 0.858 |
| NPPB | IDE | 0.83 | 0 | 0.165 | 0.852 |
| NPPB | NPR1 | 0.336 | 0 | 0.749 | 0.826 |
| NPPB | NPR2 | 0.336 | 0 | 0.731 | 0.813 |
| NPR1 | NPR2 | 0 | 0.8 | 0.922 | 0.807 |
| NPPC | NPPA | 0 | 0 | 0.804 | 0.804 |
| NPPC | NPPB | 0 | 0 | 0.788 | 0.788 |
| MYH6 | NPPA | 0 | 0 | 0.747 | 0.774 |
| NPPB | CRP | 0 | 0 | 0.733 | 0.738 |
| MYH6 | NPPB | 0 | 0 | 0.698 | 0.737 |
| NPPB | TNNI3 | 0 | 0 | 0.704 | 0.73 |
| TNNI3 | CRP | 0 | 0 | 0.705 | 0.705 |
| NPPA | TNNI3 | 0 | 0 | 0.562 | 0.591 |

NPPB = B-type natriuretic peptide

*Protein-Protein Interactions are reported from the STRING database (<https://version11.string-db.org/cgi/input.pl?sessionId=wGPwQISZgRcC&input_page_show_search=on>)

**References**

1 Szklarczyk, D. *et al.* STRING v11: protein-protein association networks with increased coverage, supporting functional discovery in genome-wide experimental datasets. *Nucleic Acids Res.* **47**, D607-D613, (2019).
